# Galectin-3 directs mitophagy in response to Parkin-/proteasome-dependent rupture of mitochondrial outer membrane

**DOI:** 10.1186/s13062-025-00692-1

**Published:** 2025-11-06

**Authors:** Pei-Han Liu, Yu-Shan Lin, Wei-Hua Chu, Wei-Tse Sun, Po-Yu Huang, Jie-rong Huang, Wei-Chung Chiang

**Affiliations:** 1 Institute of Biochemistry and Molecular Biology, College of Life Sciences, National Yang Ming Chiao Tung University, Taipei, Taiwan; 2 Program in Molecular Medicine, National Yang Ming Chiao Tung University and Academia Sinica, Taipei, Taiwan; 3 Cancer and Immunology Research Center, National Yang Ming Chiao Tung University, Taipei, Taiwan

**Keywords:** Galectin-3, PINK1/Parkin-dependent mitophagy, Mitochondrial outer membrane (OMM) rupture, Liquid-liquid phase separation (LLPS), Biomolecular condensate

## Abstract

PINK1/Parkin-dependent mitophagy is an intracellular process that selectively removes damaged, depolarized mitochondria. In this form of mitophagy, the mitochondrial outer membrane (OMM) undergoes focal rupture through a Parkin- and proteasome-dependent mechanism, which consequently results in the exposure of mitochondrial inner membrane (IMM) mitophagy receptors. The OMM rupture marks the damaged mitochondria and ensures their proper disposal. However, our understanding of the molecular events triggered by OMM rupture remains limited. Here, our proteomic study revealed that Galectin-3, a member of the β-galactoside-binding protein family, is significantly enriched in the ruptured OMM of damaged mitochondria. Galectin-3 is necessary for mitophagy, and it relocalizes from the cytosol to enclose the damaged mitochondria in response to mitophagy induction. The mitochondrial recruitment of Galectin-3 is Parkin- and proteasome-dependent, suggesting that the enclosure of mitochondria by Galectin-3 is a consequence of OMM rupture during PINK1/Parkin-mediated mitophagy. Functionally, Galectin-3 interacts with IMM protein PHB2 and recruits autophagy initiation factors ULK1 on the damaged mitochondria. Importantly, mutations in key residues that confer the liquid-liquid phase separation (LLPS) properties of Galectin-3 abrogates its mitochondrial relocalization, ULK1 recruitment, and mitophagy, suggesting that the capacity to form biomolecular condensates around the damaged mitochondria is crucial for the mitophagy function of Galectin-3. While much of the prior research on Galectin-3 focused on its extracellular functions, our findings shed light on its previously underexplored intracellular functions on the mitochondria and illuminated a novel mechanism by which Galectin-3 senses the damaged mitochondria and maintains organellar quality control.

**Supplementary Information:**

The online version contains supplementary material available at 10.1186/s13062-025-00692-1.

## Introduction

Selective autophagy of damaged mitochondria, also known as mitophagy, is a crucial degradative process essential for maintaining cellular and organismal homeostasis. As damaged mitochondria can release reactive oxygen species and mitochondrial DNA, leading to increased genotoxic stress and promoting cellular inflammation, mitophagy is important for the protection against deleterious consequences of the accumulation of damaged mitochondria [1, 2]. Studies have shown that impaired mitophagy is linked to human pathological conditions characterized by mitochondrial dysfunction [3–6], including inflammation, age-related diseases, neurodegeneration [7–10], and cancer [11–13]. Thus, the proper removal of damaged mitochondria via mitophagy plays a pivotal role in preserving organismal health [14–16].

Upon mitochondrial damage, the dissipation of the mitochondrial membrane potential leads to the stabilization and activation of serine/threonine kinase PINK1, which phosphorylates ubiquitin associated with the OMM at baseline levels and recruits E3 ubiquitin ligase Parkin to the depolarized mitochondria. These events culminate in the accumulation of phospho-ubiquitin chains, which are recognized by a set of autophagy adaptors (such as OPTN, NDP52, p62/SQSTM1, NBR1, and TAX1BP1) that interact with LC3, thereby targeting the expanding autophagic membranes to the damaged mitochondria. During this process, additional autophagy-related (ATG) proteins are also recruited to the OMM to promote the formation of autophagic membranes encapsulating the damaged mitochondria [17–19].

The current understanding of the mitophagy mechanism primarily emphasizes that the molecular signatures—ubiquitylated proteins or mitophagy receptors—on OMM enable the recognition of damaged mitochondria. However, the molecular signature of the IMM also plays a direct role in cargo recognition. During PINK1/Parkin-mediated mitophagy, the OMM can undergo proteasome- and Parkin-dependent rupture. The OMM rupture is necessary for mitophagy, as the absence of Parkin or pharmacological inhibition of proteasome abrogates the clearance of the damaged mitochondria [20, 21]. Thus, the necessity of OMM rupture for mitophagy can be explained by the presence of molecular events in which the cytoplasmic release or exposure of IMM or intermembrane space (IMS) mitophagy factors promotes the autophagic turnover of mitochondria. Supporting this hypothesis, a previous study identified an IMM protein Prohibitin 2 (PHB2) as a mitophagy receptor that biochemically interacts with LC3-II via a canonical LIR domain following OMM rupture, thereby serving as a recognition point for selective autophagy [20]. Consistent with this, a recent study also identified IMM protein MTFP1 (MTP18) as a mitophagy receptor that interacts with LC3-II in response to carbonyl cyanide m-chlorophenyl hydrazone (CCCP)-induced OMM rupture in oral cancer cells [22]. Moreover, a distinct form of OMM rupture, triggered by increased expression of E3 ubiquitin ligase AMFR or mitochondrial stress, exposes the IMM protein OPA1, which interacts with ER-phagy receptor RETREG1 to trigger reticulo-mitophagy (selective autophagy of both ER and mitochondria) [23]. Collectively, these findings illustrate that cytoplasmic exposure of specific molecular signatures of IMM is a driving force that imparts cargo selectivity in different types of mitophagy.

Compromised cellular membranes serve as a danger signal that triggers certain forms of cellular responses. In addition to mitochondria, prior studies have demonstrated that the rupture of endomembranes, including pathogen-containing endosomes, phagosomes [24], and lysosomes [25], can initiate their autophagic clearance. Thus, membrane rupture represents a common feature of organellar selective autophagy in which inner components of the organelle become topologically exposed and are “sensed” by autophagy machinery. Insights from earlier research on the selective autophagy of endomembranes indicate that the membrane damage response is a complex cellular process involving multiple mechanisms that coordinately recognize the membrane damage and mobilize core autophagy proteins to ensure the efficient disposal of organelles [26]. In contrast, our mechanistic understanding of OMM rupture within the context of PINK1/Parkin-mediated mitophagy remains limited. Thus, in this study, we conducted a proteomic study to uncover the molecular events that occur at the OMM rupture site during mitophagy.

## Results

### Identification of Galectin-3 in the proximal proteome of PHB2 during mitophagy

**Fig. 1:**
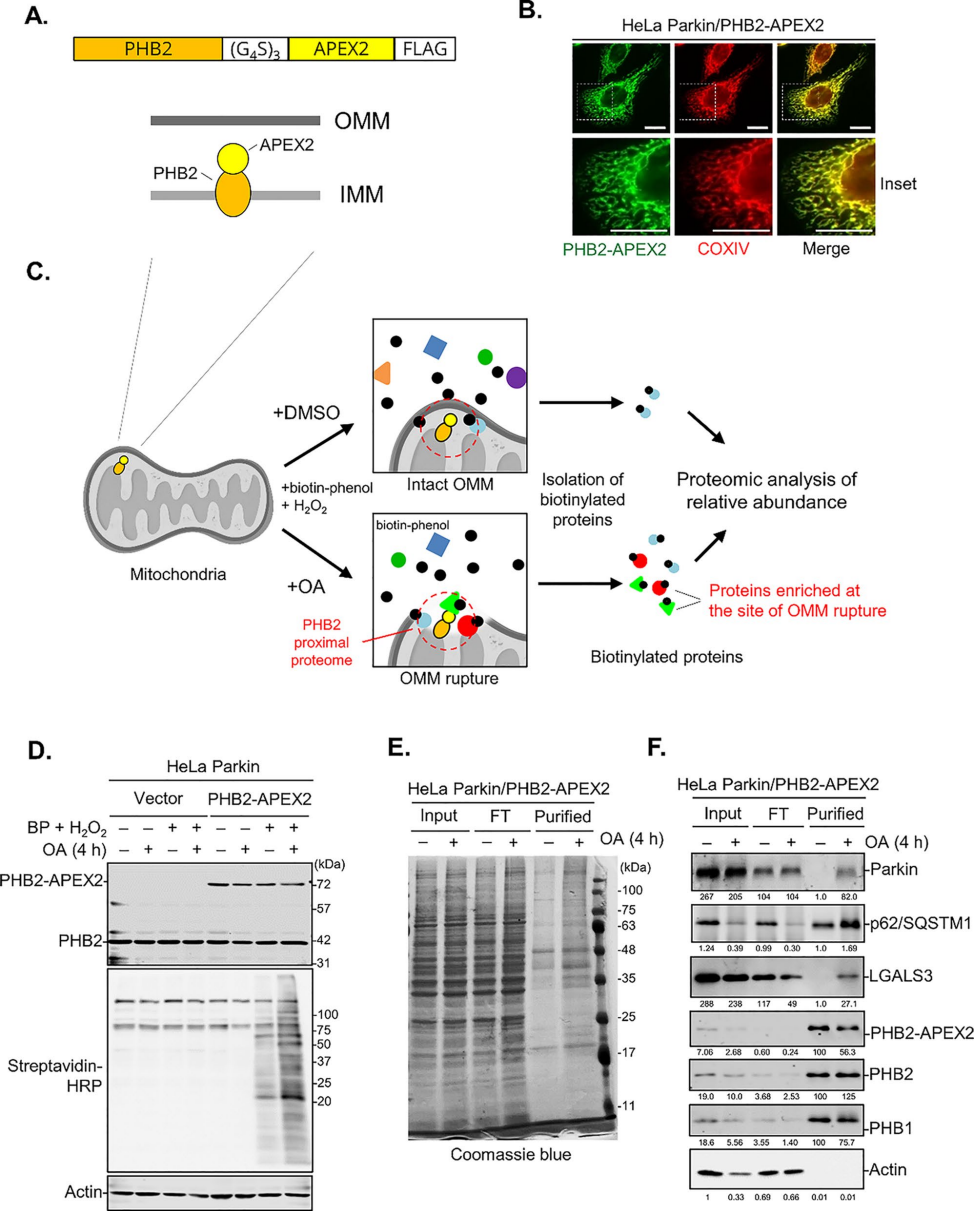

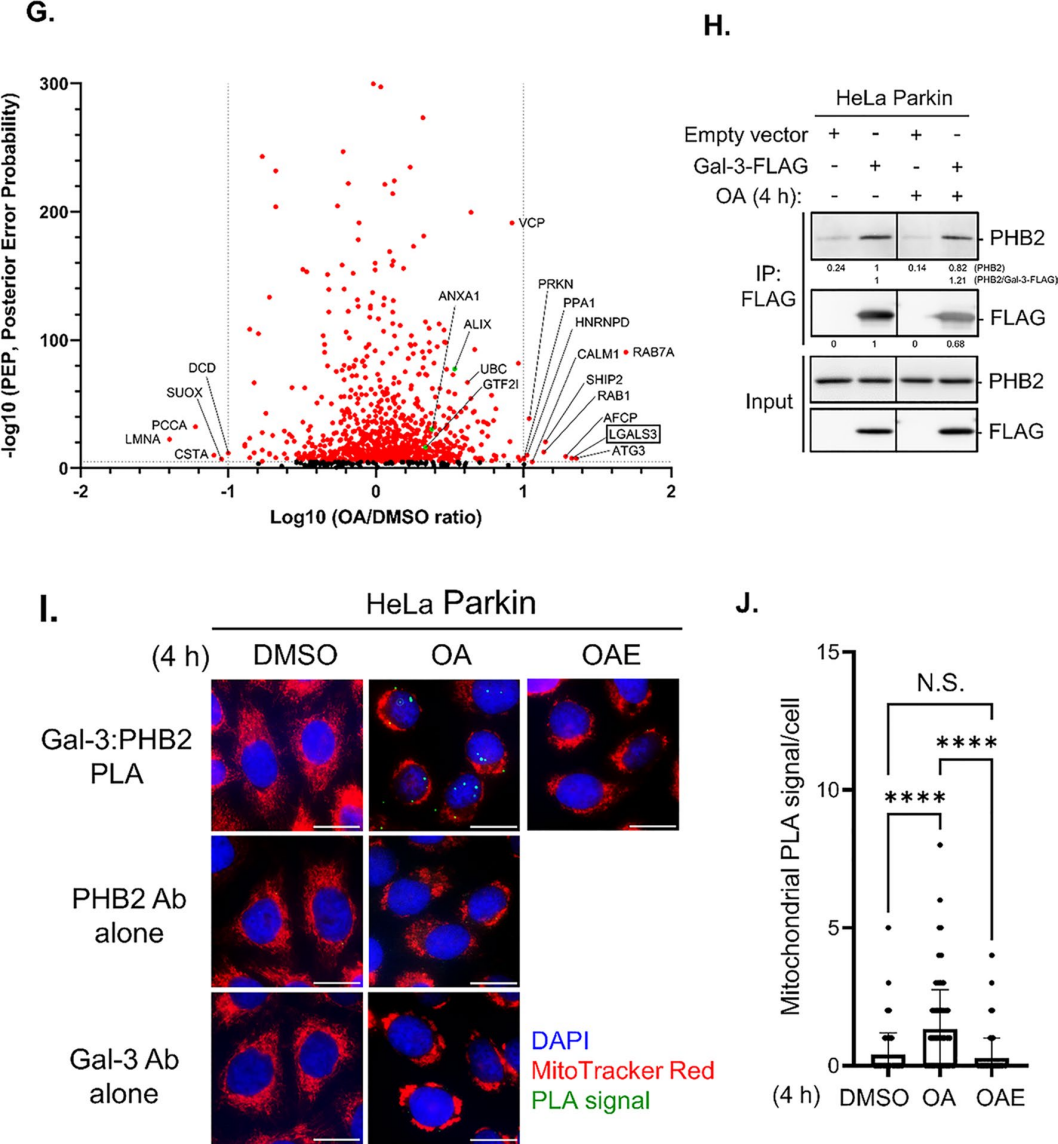
APEX2-based proximity labeling identified Galectin-3 as a PHB2-interacting protein during mitophagy**. (A)** Schematic representation of the PHB2-APEX2 expression construct used for proximity labeling. A FLAG-tagged APEX2 (yellow) is fused to the C-terminus of PHB2 (orange) via a flexible linker, positioning APEX2 on the intermembrane space face of the mitochondrial inner membrane. **(B)** Immunofluorescent images of HeLa Parkin cells expressing PHB2-APEX2-FLAG construct stained with PHB2-APEX2 (green) and COXIV (red). Scale bars, 10 μm. **(C)** HeLa Parkin cells transduced with an empty vector (vector) or PHB2-APEX2-expressing construct were treated with either DMSO or OA (oligomycin/antimycin A) for 4 h to induce mitophagy. At the end of DMSO/OA treatment, cells were briefly exposed to biotin-phenol (BP) and H_2_O_2_ to trigger the labeling proximal proteome of PHB2. **(D)** APEX2-labeled cell lysates were analyzed using Streptavidin blot. **(E)** Coomassie blue staining of APEX2-labeled cell lysates (Input), flow-through (FT), and purified biotinylated samples (Purified). (**F)** Western blot analysis on protein samples as analyzed in (**e**). Numbers shown below each panel are quantification of bands. **(G)** Volcano plot depicting the comparative spectra index between DMSO- and OA-treated samples in non-labeled proteomic analysis. The x-axis represents the ratio of spectra index (OA/DMSO), while the y-axis denotes the Posterior Error Probability (PEP), reflecting the likelihood of an incorrect peptide–spectrum match. Proteins with PEP <  1 × 10^− 5^ are marked in red, and previously reported Galectin-3 interactors are highlighted in green. Autophagy-related proteins, as well as hits showing > 10-fold changes, are labeled by gene name. **(H)** HeLa Parkin cells expressing vector or wild-type (WT) FLAG-tagged Galectin-3 (Gal-3-FLAG) were incubated with OA for 4 h and subjected to immunoprecipitation with anti-FLAG antibody. The immunopurified samples were analyzed by Western blot as shown. **(I)** Representative images of Duolink in situ PLA assay demonstrating OA-induced interaction between endogenous Galectin-3 and PHB2 in HeLa Parkin cells treated with DMSO, OA, or OA + epoxomicin (OAE). >100 cells per sample. Scale bars, 20 μm. **(J)** Quantitation of Galectin-3/PHB2 PLA signals shown in (**i**). Similar results were obtained in three independent experiments. ****P < 0.0001, One-way ANOVA with Kruskal-Wallis test

To gain a deeper understanding of the molecular dynamics at the OMM rupture site, we chose to investigate the local protein environment of PHB2, the first IMM protein reported to become exposed to the cytoplasm during mitophagy [20]. Characterizing the PHB2 proximal interactome provides a unique opportunity to uncover novel molecular mechanisms associated with OMM rupture. To do this, an enzyme-catalyzed proximity labeling technique was used to covalently label endogenous proteins that are in close proximity (less than 20 nm) to PHB2. Specifically, we genetically fused APEX2, an engineered soybean ascorbate peroxidase [27] to the C-terminal of PHB2 (Fig. 1A) and transfected the PHB2-APEX construct into HeLa cells stably expressing Parkin (designated as HeLa Parkin). The immunofluorescent staining confirmed efficient mitochondrial targeting of PHB2-APEX2 fusion protein (Fig. 1B).

We first evaluated the efficacy of this approach in capturing the PHB2 interactome. HeLa Parkin/PHB2-APEX2 cells were incubated with either DMSO or OA (mitochondrial toxins that induce PINK1/Parkin-dependent mitophagy) for 4 h, a timeframe during which the early events of mitophagy (including OMM rupture) are initiated but substantial mitochondrial clearance has not yet occurred (Fig. 1C) [20]. This was followed by a brief exposure to H_2_O_2_ and biotin-phenol (BP, a substrate of APEX2) to trigger the biotinylation of proximal proteins of PHB2-APEX2. Subsequent streptavidin-HRP blot analysis of cell lysates revealed that a large number of endogenous proteins were tagged with biotin. The biotin labeling almost exclusively depends on the expression of PHB2-APEX2 and the incubation with H_2_O_2_ and BP (Fig. 1D). Importantly, OA treatment led to the appearance of numerous unique bands, indicating that the proximal proteome of PHB2 undergoes substantial alteration during mitophagy. Consistently, Coomassie blue staining of the purified biotinylated samples also showed a similar trend (Fig. 1E). Western blot validation of the isolated biotinylated samples revealed that Parkin was absent in the PHB2 proximal proteome under basal conditions, but significantly enriched upon OA treatment (Fig. 1F). This result aligned with a recent report in which the interaction between Parkin and PHB2 occurs exclusively during mitophagy [28]. In addition, we also observed an OA-induced enrichment of p62/SQSTM1, a ubiquitin-binding autophagy receptor that has recently been shown to interact with PHB2 during bile acid-induced mitophagy [29]. Moreover, APEX2 also tagged prohibitin complexes (PHB1 and PHB2), demonstrating that the proximity labeling technique reliably captures native protein-protein interactions on the IMM. These data affirmed the effectiveness and specificity of APEX2-based proximity labeling.

We subsequently conducted a label-free proteomic analysis on the isolated biotinylated protein samples to identify new molecules that are enriched upon mitophagy induction. Normalized spectra indexes (the relative abundance of a protein) revealed a significant enrichment of 105 proteins in OA-treated cells, including 55 hits that were exclusively detected in OA-treated samples (Table 1 and S1). Notably, Parkin (PRKN) and ubiquitin (UBC) —both previously reported to interact with or conjugate to PHB2 during mitophagy [28, 30] —showed a marked OA-dependent increase in the proximal proteome of PHB2 (Fig. 1G). Among these candidate molecules were several known autophagy and mitophagy factors (ATG3, RAB1, RAB7A, and VCP), indicating that these proteins are recruited to the vicinity of OMM rupture sites during mitophagy. Notably, spectral count analysis revealed a 21-fold increase of LGALS3 (known as Galectin-3), in mitophagy-inducing conditions (Table S1, highlighted in red), indicating a marked enrichment of Galectin-3 in the PHB2 proximal proteome during mitophagy. Consistent with this, three known interactors of Galectin-3 (ALIX, ANXA1, and GTF2I; Fig. 1G, shown in green) were also detectable in the PHB2 proximal interactome during mitophagy, though their OA-induced enrichment was modest (Table S1). Subsequent Western blot analysis of APEX2-labeled protein samples revealed that Galectin-3 was undetectable in basal conditions but markedly increased in the PHB2 proximal proteome upon mitophagy induction (Fig. 1F).

**Table 1:**
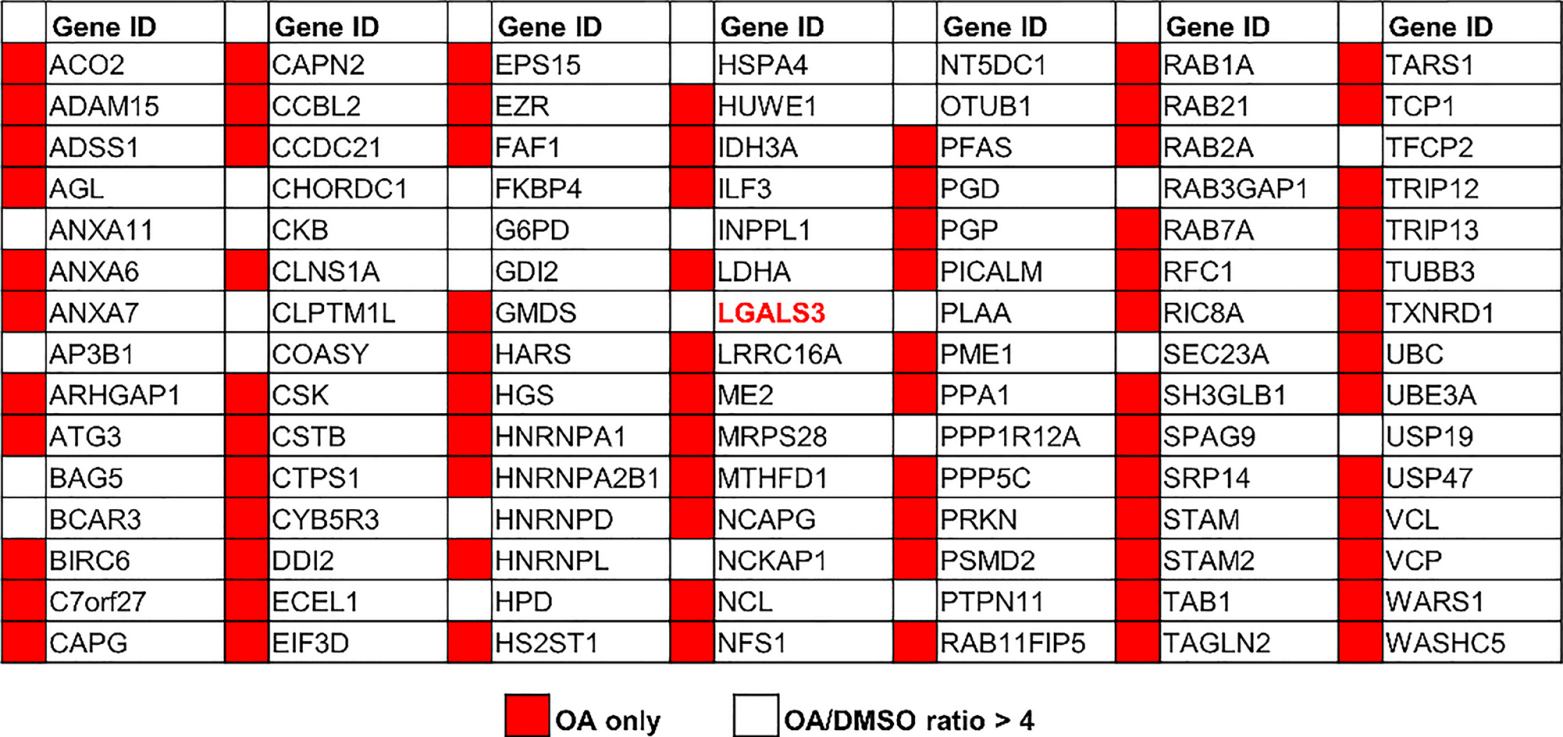
Select mitophagy-regulated PHB2 proximal proteins identified by APEX2-based proximity labeling. Red, proteins detected exclusively in OA-treated samples; White, proteins showing > 4-fold enrichment in OA-treated samples compared with DMSO controls. A posterior error probability (PEP) cutoff of 1 × 10⁻⁵ was applied

Galectin-3 is a member of the beta-galactoside-binding protein family that recognizes glycoproteins or glycolipids on the luminal or exofacial side of the endomembranes or plasma membrane. It plays a significant role in various cellular processes, including cell adhesion, cell growth, cell cycle, and apoptosis [31, 32]. Physiologically, increased Galectin-3 is highly relevant to the pathogenesis of a wide range of human diseases, including cancer, fibrosis, and chronic inflammation [33]. Previous studies reported that Galectin-3 is involved in the homeostatic response to damaged endomembranes, including lysosome, endosome, or intracellular pathogens undergoing endosomal escape [26, 31]. Functionally, Galectin-3 aggregates at sites of endomembrane rupture and recruits autophagy machinery, promoting the autophagic turnover of the target organelle or ESCRT-mediated membrane repair [34, 35]. Thus, the identification of Galectin-3 in the proximal proteome of PHB2 in cells undergoing mitophagy suggested that it may play a role in the clearance of damaged, OMM-ruptured mitochondria. However, the specific function of Galectin-3 in mitophagy remains unknown.

### Galectin-3 interacts with PHB2 during OMM rupture

The initial identification of Galectin-3 in the PHB2 proximal proteome suggested that Galectin-3 is likely a binding protein of PHB2. To investigate this, we performed co-immunoprecipitation experiments in HeLa Parkin cells expressing FLAG-tagged Galectin-3. Western blot analysis of immunoprecipitated samples revealed an interaction of PHB2 and Galectin-3 in both DMSO and OA-treated samples (Fig. 1H). However, the binding of Galectin-3 to PHB2 is not specific to mitophagy-inducing conditions, which appeared to be contradictory to our proximity labeling data in which the proximal interaction between PHB2 and Galectin-3 occurs almost exclusively during mitophagy (Fig. 1F). We reasoned that this discrepancy may stem from the non-native interaction of Galectin-3 and PHB2 in cell homogenate in which the mitochondrial membranes are disrupted. To further elucidate this, we performed an in situ proximity ligation assay (PLA) to probe the interaction between Galectin-3 and PHB2. In HeLa Parkin cells, we detected minimal PLA signals in cells treated with DMSO, indicative of a basal interaction between Galectin-3 and PHB2. Upon mitophagy induction with OA, there was a substantial increase in the number of mitochondria-localized PLA puncta (Fig. 1I and J). The staining with either anti-PHB2 or anti-Galectin-3 antibody did not result in significant PLA signals in both DMSO- or OA-treated conditions, confirming the specificity of PLA in detecting Galectin-3/PHB2 interaction. Importantly, the inhibition of OMM rupture with epoxomicin hindered the formation of PLA signals, suggesting that the topological exposure of IMM is required for Galectin-3/PHB2 interaction during mitophagy.

### Galectin-3 is required for mitophagy

**Fig. 2:**
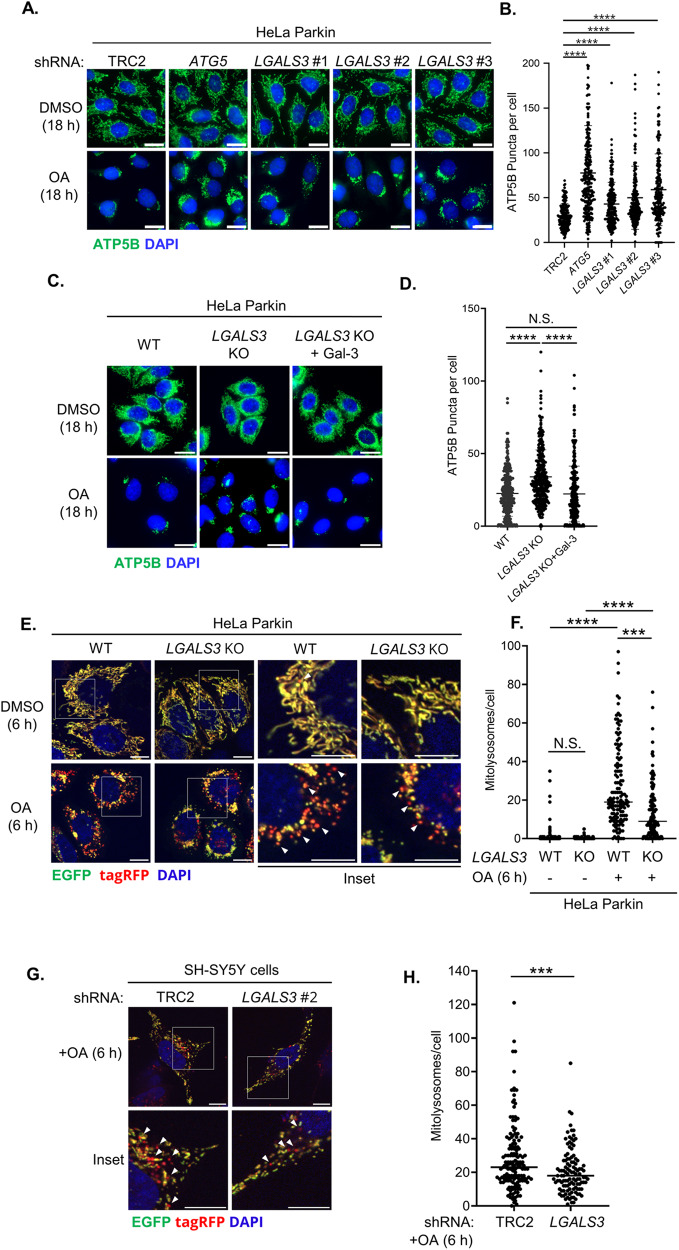
Galectin-3 is required for mitophagy**. (A)** Representative immunofluorescent images of HeLa Parkin cells expressing control shRNA (TRC2) or shRNA targeting *LGALS3* or *ATG5* incubated with either DMSO or OA for 18 h, and the samples were immunofluorescently stained for mitochondrial marker ATP5B (green). **(B)** Quantitation of ATP5B puncta in (**a**). **(C)** Representative images of HeLa Parkin wild-type (WT), Galectin-3 knockout (*LGALS3* KO), or *LGALS3* KO with single-copy knock-in of Galectin-3 (*LGALS3* KO + Gal-3) treated with either DMSO or OA for 18 h and stained for ATP5B (green). **(D)** Quantification of ATP5B puncta in (**c**). Scale bars, 20 μm. >150 cells per sample. Similar results were obtained in three independent experiments. ****P < 0.0001, N.S. non-significant, One-way ANOVA with Kruskal-Wallis test. **(E)** Representative fluorescent micrographs of HeLa Parkin wild-type (WT) or *LGALS3* KO cells expressing COX8-EGFP-tagRFP-PEST reporter were incubated with either DMSO or OA for 6 h. Scale bars, 20 μm. **(F)** Quantitation of mitolysosomes (EGFP^-^/tagRFP^+^ puncta) shown in (**e**). One-way ANOVA with Kruskal-Wallis test. **(G)** Representative images of SH-SY5Y expressing either TRC2 (control) or *LGALS3* shRNA #2 and mitophagy flux reporter. Samples were treated with either DMSO or OA for 6 h. Scale bars, 10 μm. **(H)** Quantitation of mitolysosomes in (**g**). >100 cells were analyzed per sample. Similar results were obtained in three independent experiments. N.S. non-significant, ****P < 0.0001, ***P < 0.001. Mann-Whitney test

The proximal interaction between Galectin-3 and PHB2 induced by OA strongly suggested that Galectin-3 may participate in the damage response involving mitophagy. To test this, we evaluated whether Galectin-3 is required for the clearance of damaged mitochondria. We generated HeLa Parkin cells that express shRNA targeting *LGALS3* and monitored the OA-induced elimination of ATP5B, a mitochondrial protein. Immunofluorescent analyses showed that the depletion of core autophagy gene *ATG5* resulted in impaired clearance of mitochondria, and the depletion of Galectin-3 with three different shRNAs (Fig. S1A) hindered OA-induced mitochondrial clearance to an extent similar to the *ATG5* knockdown (Fig. 2A and B). Likewise, the knockout (KO) of Galectin-3 resulted in clearance defects, which can be fully rescued by a single copy knock-in of Galectin-3 cDNA at AAVS1 locus (a well-recognized safe harbor for transgenic knock-in) (Fig. 2C–D and S1B). These findings suggest that Galectin-3 is required for the removal of damaged mitochondria. Additionally, Galectin-3 depletion minimally affected LC3-II conversion or p62 degradation under basal or mitophagy-inducing conditions (Fig. S2A), and the steady-state levels of core autophagy proteins, including ATG5, ATG7, and BECN1, remained largely unchanged (Fig. S2B). These observations indicated that defective mitochondrial clearance in Galectin-3-deficient cells is unlikely to be attributed to the impairment of the core autophagy processes.

To rigorously investigate the role of Galectin-3 in mitophagy, we performed mitophagy flux assays to determine the requirement of Galectin-3 for the lysosomal delivery of mitochondria by using a tandem bifluorescent mitophagy flux reporter (2xCOX8-EGFP-tagRFP-PEST). Our data showed that *LGALS3* KO resulted in a diminished number of mitolysosomes (Fig. 2E and F), suggesting that Galectin-3 is necessary for the lysosomal delivery of damaged mitochondria. To further examine the mitophagy function of Galectin-3 in neuronal mitophagy, we performed mitophagy flux assays in neuroblastoma cell line SH-SY5Y, which expresses endogenous levels of Parkin. shRNA-mediated knockdown of Galectin-3 (Fig. S1C) resulted in a mild decrease in the number of mitolysosomes in SH-SY5Y following OA treatment (Fig. 2G and H), indicating that Galectin-3 is also required for mitophagy in neuronal cells with physiologically relevant levels of Parkin expression. Taken together, these data revealed that Galectin-3 is a bona-fide mitophagy factor.

### Galectin-3 relocalizes to the damaged mitochondria upon Parkin- and proteasome-dependent OMM rupture

**Fig. 3:**
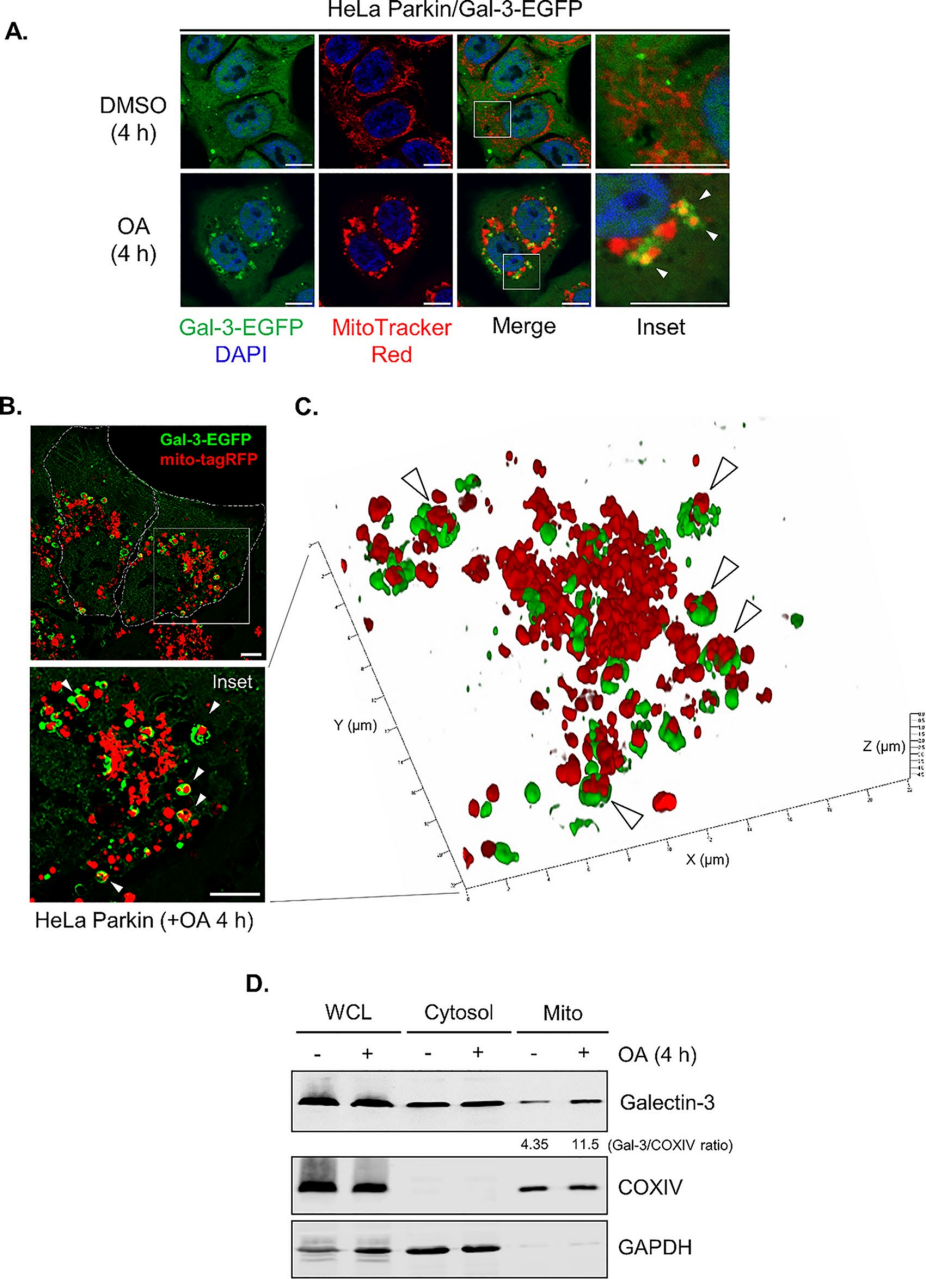
Galectin-3 is recruited to the mitochondria during mitophagy.** (A)** Representative images of HeLa Parkin cells stably expressing EGFP-tagged Galectin-3 (Gal-3-EGFP, green) stained with MitoTracker Red (red) and treated with either DMSO or OA for 4 h. Scale bars, 10 μm. **(B)** Representative SIM images of HeLa Parkin cells expressing Gal-3-EGFP and mitochondria-targeted tagRFP (mito-tagRFP) revealed that mitochondria is encapsulated in Galectin-3-positive structures, as indicated by white arrows. White dotted lines denote cell boundaries. Scale bars, 5 μm. **(C)** 3D reconstruction of SIM images shown in **(b)**. Arrows indicate the mitochondria surrounded by Galectin-3. **(D)** Western blot analysis of the isolated mitochondrial whole cell lysates (WCL), cytosolic (Cytosol), and mitochondrial (Mito) fractions of HeLa Parkin cells treated with either DMSO or OA for 4 h. COXIV, mitochondrial marker. GAPDH, cytosol marker. Similar results were obtained in three independent experiments

The enrichment of Galectin-3 in the PHB2 proximal proteome under mitophagy-inducing conditions (Fig. 1 F and G) suggests that Galectin-3 may relocalize to the damaged mitochondria to facilitate mitophagy. To examine this, we stably expressed EGFP-tagged Galectin-3 (Galectin-3-EGFP) in HeLa Parkin Galectin-3 KO cells and monitored the subcellular distribution of Galectin-3-EGFP under both basal and mitophagy-inducing conditions. Fluorescent microscopy revealed that under basal conditions, Galectin-3-EGFP signals were diffused and cytoplasmic, occasionally forming random cytoplasmic puncta that rarely overlap with the mitochondria. Upon a 4-hour treatment with OA, there was a marked increase in the Galectin-3-positive signals surrounding the clustered mitochondria in the perinuclear regions (Fig. 3A). Time course analysis also demonstrated a progressive increase in mitochondria-localized Galectin-3 throughout the OA treatment (Fig. S3A–C). Consistent with this, treatment with mitochondrial uncoupler CCCP also induced mitochondrial clustering of Galectin-3 (Fig. S3D–F), indicating that the mitochondrial relocalization of Galectin-3 is due to the induction of PINK1/Parkin-dependent mitophagy.

To better characterize this, we utilized super-resolution structured illumination microscopy (SIM) to visualize the Galectin-3-positive puncta formed during mitophagy. Three-dimensional reconstruction of SIM images revealed that both smaller and larger perinuclear-compacted mitochondria can be partially or completely encapsulated within Galectin-3-positive structures in cells treated with OA (Fig. 3B and C), indicating that Galectin-3 encloses the damaged mitochondria in response to mitochondrial damage. Consistent with these findings, Western blot analysis of the isolated mitochondria fractions of HeLa Parkin cells revealed that Galectin-3 is present at a minimal level on the mitochondria under basal conditions, and OA-induced mitophagy induction led to a significant increase of mitochondria-associated Galectin-3 (Fig. 3D). Together, these results clearly demonstrated that Galectin-3 is clustered to the vicinity of the damaged mitochondria during mitophagy.

**Fig. 4:**
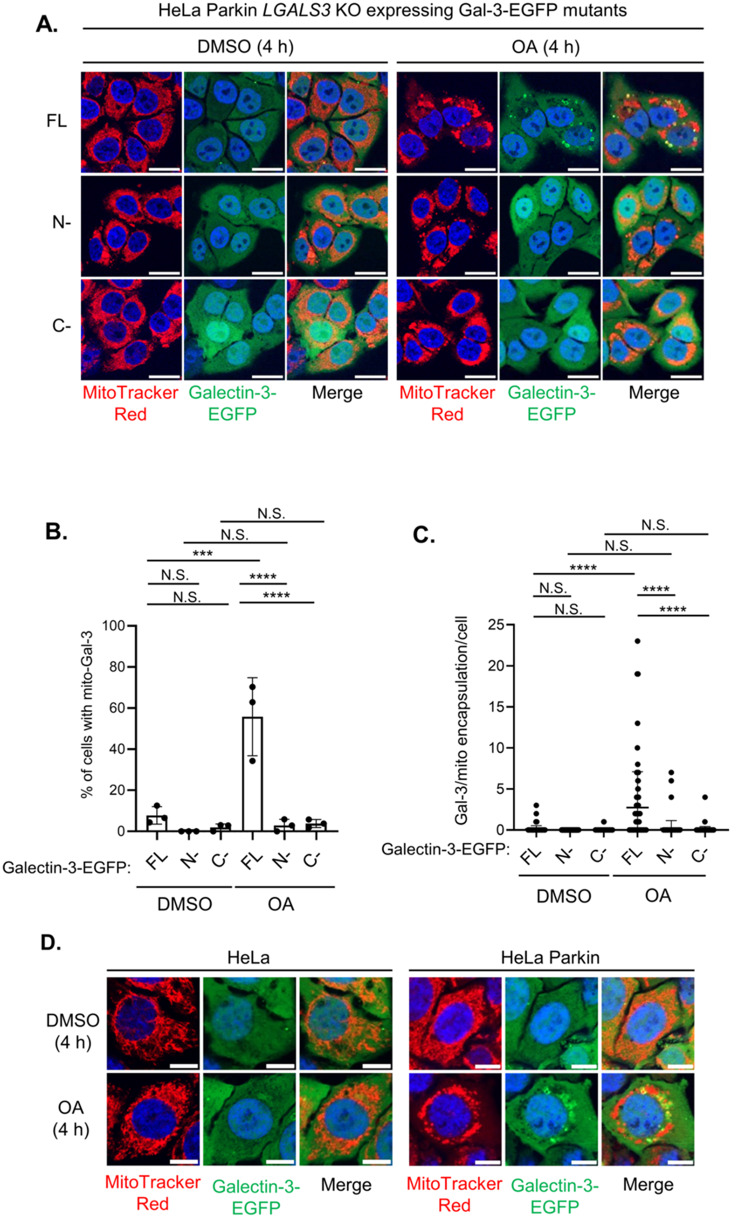

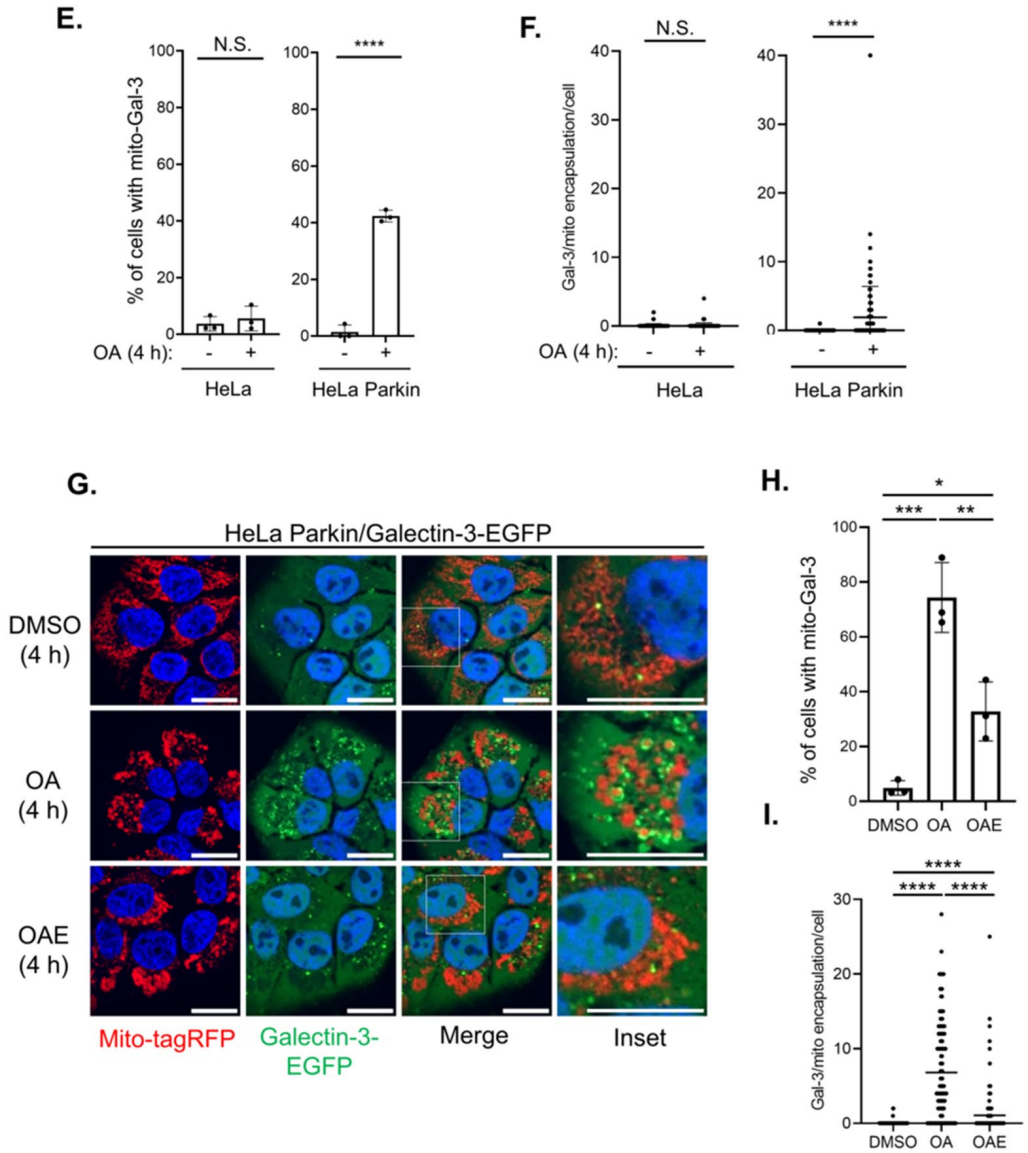
Mitophagy-regulated Galectin-3 recruitment is Parkin- and proteasome-dependent.** (A)** Representative images of HeLa Parkin *LGALS3* KO cells re-expressing full-length (FL), N-terminal (N-), or C-terminal (C-) fragments of Galectin-3-EGFP were treated as indicated for 4 h. Scale bars, 20 μm. **(B)** Quantification of the percentage of cells with mito-Gal-3. One-way ANOVA with Welch’s test. **(C)** The number of Galectin-3-positive structures encapsulating the mitochondria per cell. One-way ANOVA with Kruskal-Wallis test. **(D)** Representative images of HeLa or HeLa Parkin cells expressing Galectin-3-EGFP (green) treated with MitoTracker Red (red) were treated with either DMSO or OA for 4 h. Scale bars, 10 μm. **(E)** Quantification of the percentage of cells with Galectin-3 encapsulating the mitochondria (mito-Gal-3). Welch’s t-test. **(F)** Quantitation of Galectin-3-positive structures encapsulating the mitochondria per cell. Mann-Whitney test. **(G)** Representative images of HeLa Parkin cells expressing Galectin-3-EGFP and mito-tagRFP were treated with DMSO, OA, or OA + epoxomicin (OAE) for 4 h before the analysis. **(H)** Quantification of the percentage of cells with mito-Gal-3. One-way ANOVA with Welch’s test. **(I)** The number of Galectin-3-positive structures encapsulating the mitochondria per cell. One-way ANOVA with Kruskal-Wallis test. N.S. non-significant, *P < 0.05, **P < 0.01, ***P < 0.001, ****P < 0.0001 for all tests. Similar results were obtained in three independent experiments

Galectin-3 contains an N-terminal intrinsically disordered region (IDR) that is responsible for the self-association and a carbohydrate-recognition domain that normally interacts with glycans or proteins. To determine the functional domain(s) involved in the mitochondrial recruitment of Galectin-3, we expressed EGFP fusion of either Galectin-3 N-terminal (1-121 a.a.) or C-terminal (122–250 a.a.) fragment in HeLa Parkin cells and monitor their mitochondrial recruitment. Both N- and C-terminal fragments of Galectin-3 remained cytosolic and did not encapsulate the mitochondria during mitophagy (Fig. 4A–C), suggesting that both domains are involved in the mitochondrial clustering of Galectin-3 during mitophagy.

To further determine whether the mitochondrial recruitment of Galectin-3 is a consequence of the activation of PINK1/Parkin-mediated mitophagy, we evaluated Galectin-3 recruitment in HeLa cells, in which endogenous Parkin expression is absent. As opposed to the pronounced Galectin-3 clustering observed in HeLa Parkin cells, the mitochondrial Galectin-3-EGFP signals was absent in HeLa cells (Fig. 4D–F), indicating that mitochondrial relocalization of Galectin-3 occurs downstream of Parkin activation. Because Galectin-3 was identified in the PHB2 proximal proteome during OMM rupture, we next sought to determine whether the break of OMM is necessary for Galectin-3 recruitment. Inhibition of OMM rupture with proteasome inhibitor epoxomicin drastically reduced the size and the number of Galectin-3-positive puncta and prevented the encapsulation of mitochondria by Galectin-3 (Fig. 4G–I), suggesting that the exposure of IMM is a prerequisite for the encapsulation of mitochondria by Galectin-3. Collectively, these results indicate that Galectin-3 may play a role in sensing the damaged mitochondria through a mechanism that requires Parkin- and proteasome-dependent OMM rupture for mitophagy.

In light of our identification that Galectin-3/PHB2 proximal interaction during mitophagy, we hypothesized that the interaction of Galectin-3 with PHB2 may be responsible for its recruitment. Surprisingly, siRNA-mediated knockdown of PHB2 did not impact the mitochondrial recruitment of Galectin-3 (Fig. S3G–I), indicating that while the interaction between Galectin-3 and PHB2 occurs during OMM rupture, it may not be critical to the mitochondrial recruitment of Galectin-3.

### Galectin-3 is required for efficient recruitment of ULK1 during mitophagy

**Fig. 5:**
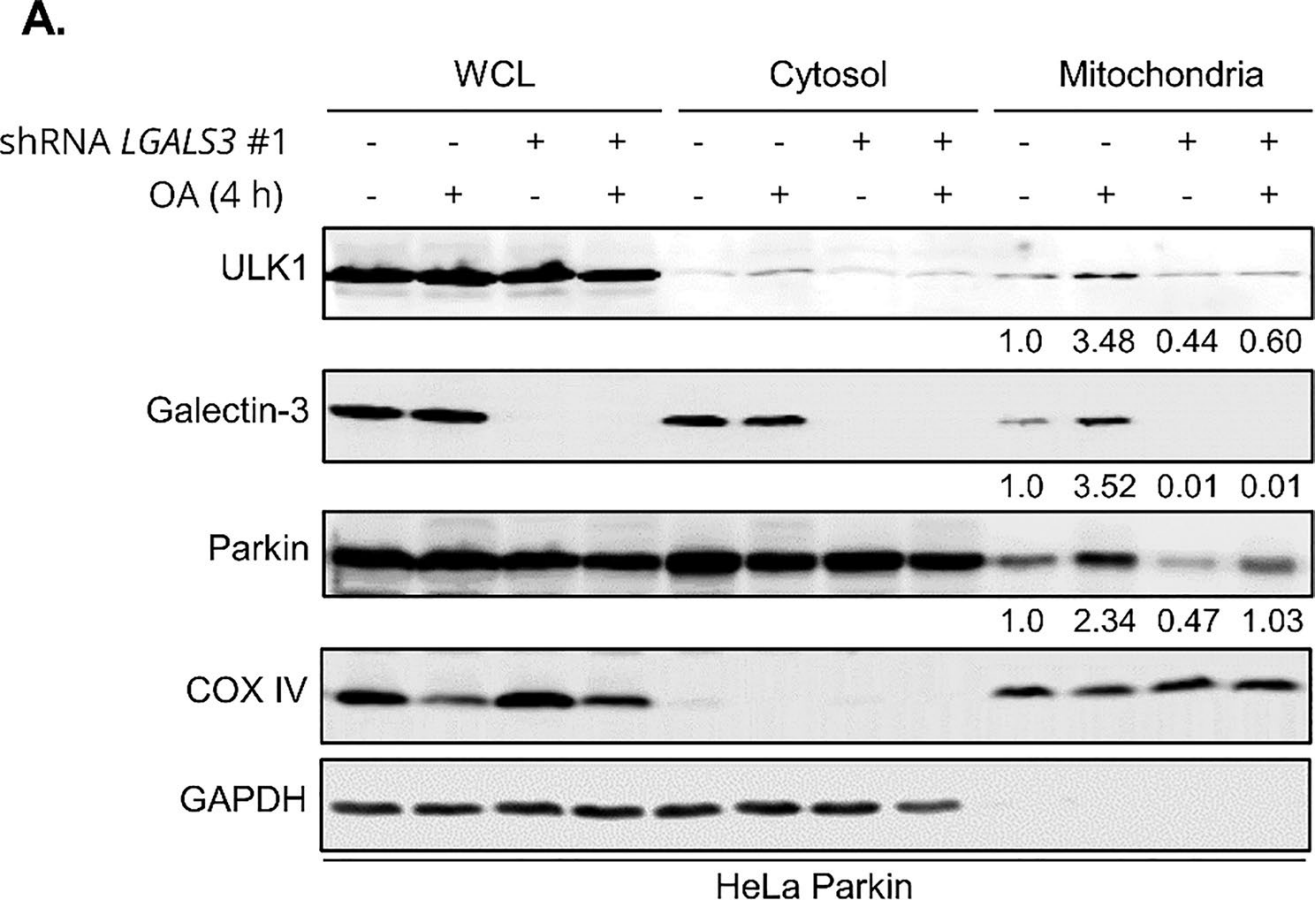
Galectin-3 is required for the mitochondrial recruitment of ULK1 during mitophagy. ** (A)** Western blot analysis of whole cell lysate (WCL), Cytosolic, and mitochondrial fractions from HeLa Parkin cells expressing control (TRC2) or *LGALS3* shRNA and incubated with DMSO or OA for 4 h

A previous study on selective autophagy revealed that Galectin-3 is relocalized to the ruptured endomembrane, where it forms a complex with autophagy initiation protein ULK1 and E3 ubiquitin ligase TRIM16 that functions as a platform to mobilize additional core autophagy factors, supporting growth of autophagosome around the ruptured endomembranes. Given the identification of Galectin-3 in mitophagy, we postulated that Galectin-3 may function similarly to promote the clearance of damaged mitochondria. Consistent with this notion, Western blot analysis of the isolated mitochondrial fractions (Fig. 5A) indicated that in the wild-type cells, levels of mitochondrial ULK1 are increased during OA-induced mitophagy, whereas the loss of Galectin-3 abrogated OA-induced accumulation of ULK1 on the mitochondria. However, we were not able to reliably detect the mitochondrial recruitment of TRIM16 (data not shown), thus its potential contribution in Galectin-3-mediated mitophagy could not be fully evaluated. The depletion of Galectin-3 did not hinder the OA-induced increase of Parkin on the mitochondria, reinforcing our previous finding that Galectin-3 functions downstream of Parkin. Together, these findings suggest that the clustering of Galectin-3 is likely to provide a structural platform necessary for ULK1 recruitment during mitophagy.

### The ability of self-association is essential for the mitophagy function of Galectin-3

The formation of biomolecular condensate is a process that compartmentalizes, segregates, or concentrates certain biomacromolecules into gel-like, membrane-less structures. Such a process is shown to play a critical regulatory role in selective autophagy, including the initiation of autophagy, regulation of TORC1, and the promotion of stress granule protein cargos for autophagic sequestration [36]. Recent studies have reported that Galectin-3 forms biomolecular condensates via liquid-liquid phase separation (LLPS) [37–39], and this property influences its oligomerization in vivo [37] or agglutination in vitro [38]. Thus, our discovery that Galectin-3 concentrates around the mitochondria during mitophagy raised a compelling question: does the mitochondrial recruitment of Galectin-3 involve the formation of biomolecular condensate, and does this process underlie the mitophagy function of Galectin-3? Previous studies have demonstrated that the N-terminal intrinsically disordered region (1-121 a.a.) of Galectin-3 is critical for the formation of biomolecular condensate [38]. This region is rich in tryptophan and tyrosine, which imparts liquid-liquid phase separation properties. The mutation of all tryptophan and tyrosine residues to Glycine (WY/G) disrupts the self-association of Galectin-3 and prevents the formation of biomolecular condensate in vitro [38]. To examine whether the formation of biomolecule condensate of Galectin-3 is important for mitophagy, we utilized the WY/G mutant as a model to assess the requirement of self-association for the mitophagy function of Galectin-3.

**Fig. 6:**
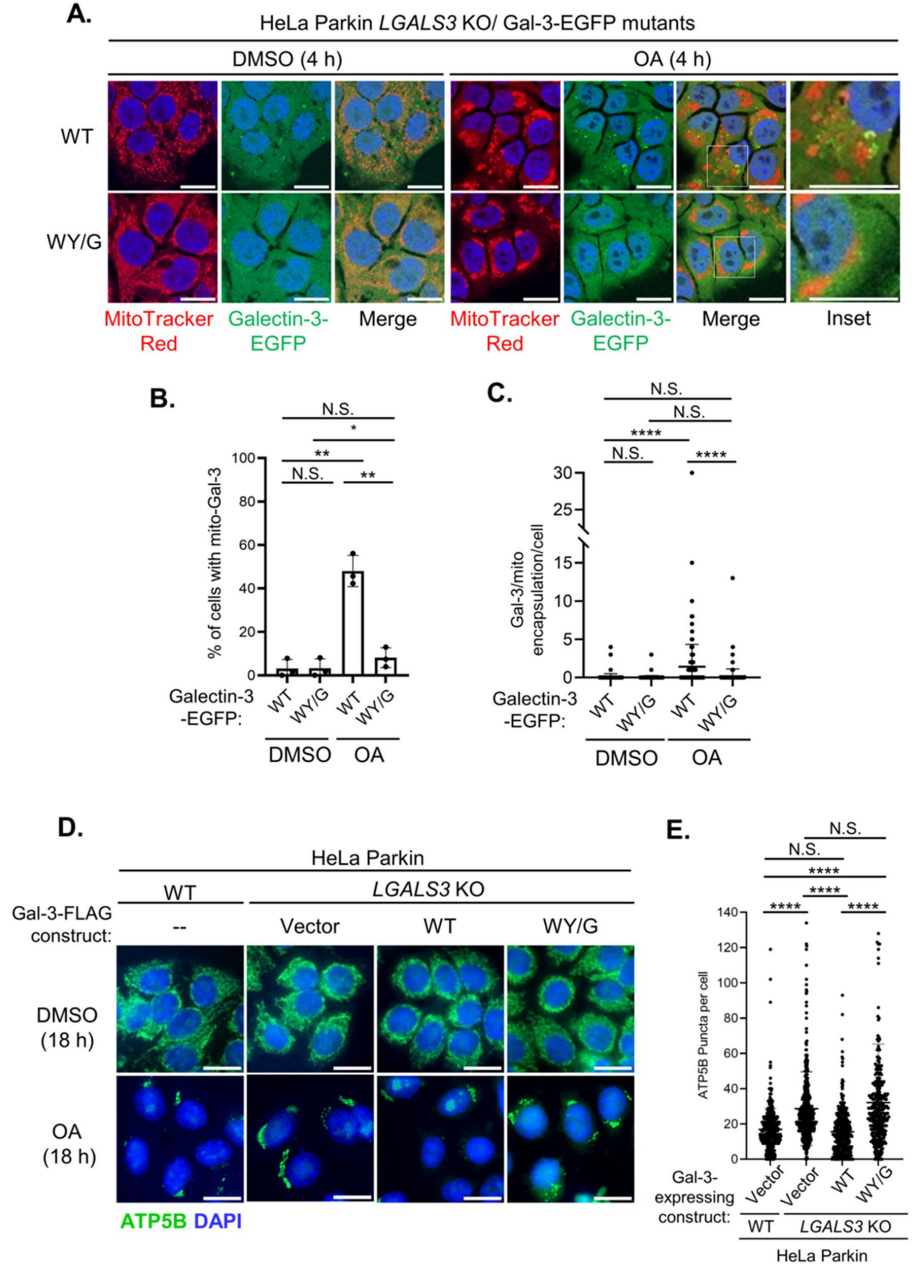

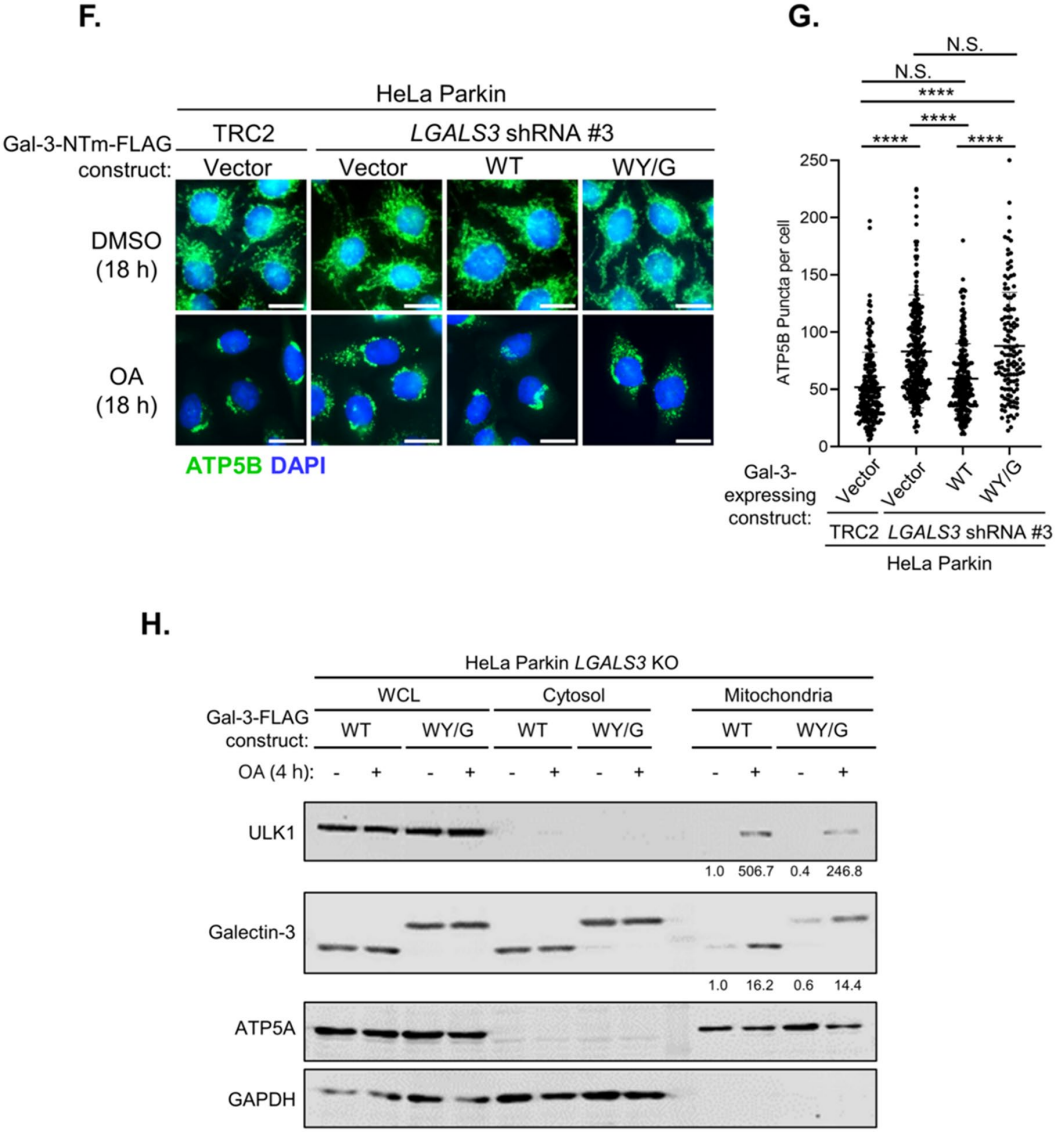
The property of Galectin-3 forming biomolecular condensates is essential for its mitophagy function. **(A)** Representative images of HeLa Parkin *LGALS3* KO cells expressing wild-type (WT), or WY/G mutant of Galectin-3-EGFP. Cells were treated with either DMSO or OA for 4 h and subjected to immunofluorescent analysis. **(B)** Quantification of the percentage of cells with mitochondrial recruitment of Galectin-3 (mito-Gal-3). One-way ANOVA with Welch’s test. **(C)** Quantification of the number of Gal-3/mito encapsulation per cell. One-way ANOVA with Kruskal-Wallis test. >100 cells per sample. **(D)** Representative immunofluorescent images of wild-type (WT) or *LGALS3* KO HeLa Parkin cells expressing Galectin-3 expressing constructs as indicated. Cells were treated with either DMSO or OA for 18 h. **(E)** Quantitation of cytoplasmic ATP5B puncta of OA-treated samples in **(d)**. >100 cells per sample. One-way ANOVA with Kruskal-Wallis test. **(F)** Representative immunofluorescent images of control (TRC2) or *LGALS3* shRNA-expressing HeLa Parkin reconstituted with shRNA non-targeted (NTm) constructs encoding wild-type (WT) or WY/G mutants of Galectin-3. Cells were subjected to treatment with DMSO or OA for 18 h. **(G)** Quantitation of cytoplasmic ATP5B puncta OA-treated samples in **(f)**. >100 cells per sample. One-way ANOVA with Kruskal-Wallis test. **(H)** Western blot analysis of whole cell lysate (WCL), Cytosolic, and mitochondrial fractions from HeLa Parkin *LGALS3* shRNA knockdown cells expressing either wild-type (WT) or WY/G mutant of Galectin-3. Cells were incubated with DMSO or OA for 4 h and subjected to fractionation. N.S. non-significant, *P<0.05, **P<0.01, ****P<0.0001 for all tests. Scale bars, 20 µm. Similar results were obtained in three independent experiments.

We expressed either the wild-type or WY/G mutant of Galectin-3-EGFP in Galectin-3 KO cells and monitored OA-induced mitochondrial clustering of Galectin-3. Under basal conditions, both the wild-type and self-association-deficient WY/G mutant exhibited a homogenous cytosolic distribution and did not form any distinct structure. Upon treatment with OA, the wild-type Galectin-3 encapsulates the damaged mitochondria, but the WY/G mutant remained completely cytosolic (Fig. 6A–C). This observation indicates that the propensity of self-association is required for the mitochondrial clustering of Galectin-3 during mitophagy. In support of this, the reconstitution of wild-type Galectin-3 in either Galectin-3 KO cells (Fig. S1D) or knockdown cells (Fig. S1E) effectively rescued the defects in mitochondrial clearance (Fig. 6D–G); in contrast, re-expression of the WY/G mutant failed to restore clearance. Also, Western blot analysis of cellular fractions revealed that the WY/G mutation impaired mitochondrial recruitment of ULK1 during mitophagy, although it did not completely abolish ULK1 localization (Fig. 6H, **top panel**). The reduced efficiency of ULK1 recruitment suggested that Galectin-3 self-association is important for ULK1 recruitment and subsequent mitochondrial clearance. Notably, the Galectin-3 WY/G mutant still displayed an OA-induced enrichment in the mitochondrial fraction, albeit to a reduced extent (Fig. 6H, **middle panel**). This differs from our immunofluorescent observations (Fig. 6A–C), which showed that WY/G failed to form cluster around the damaged mitochondria. Thus, it appears that WY/G mutation does not abolish the engagement of Galectin-3 with damaged mitochondria but specifically impairs the encapsulation of mitochondria. Collectively, these data indicated that the capacity of Galectin-3 to form biomolecular condensate is critical for its clustering around the mitochondria and mitophagy function.

**Fig. 7:**
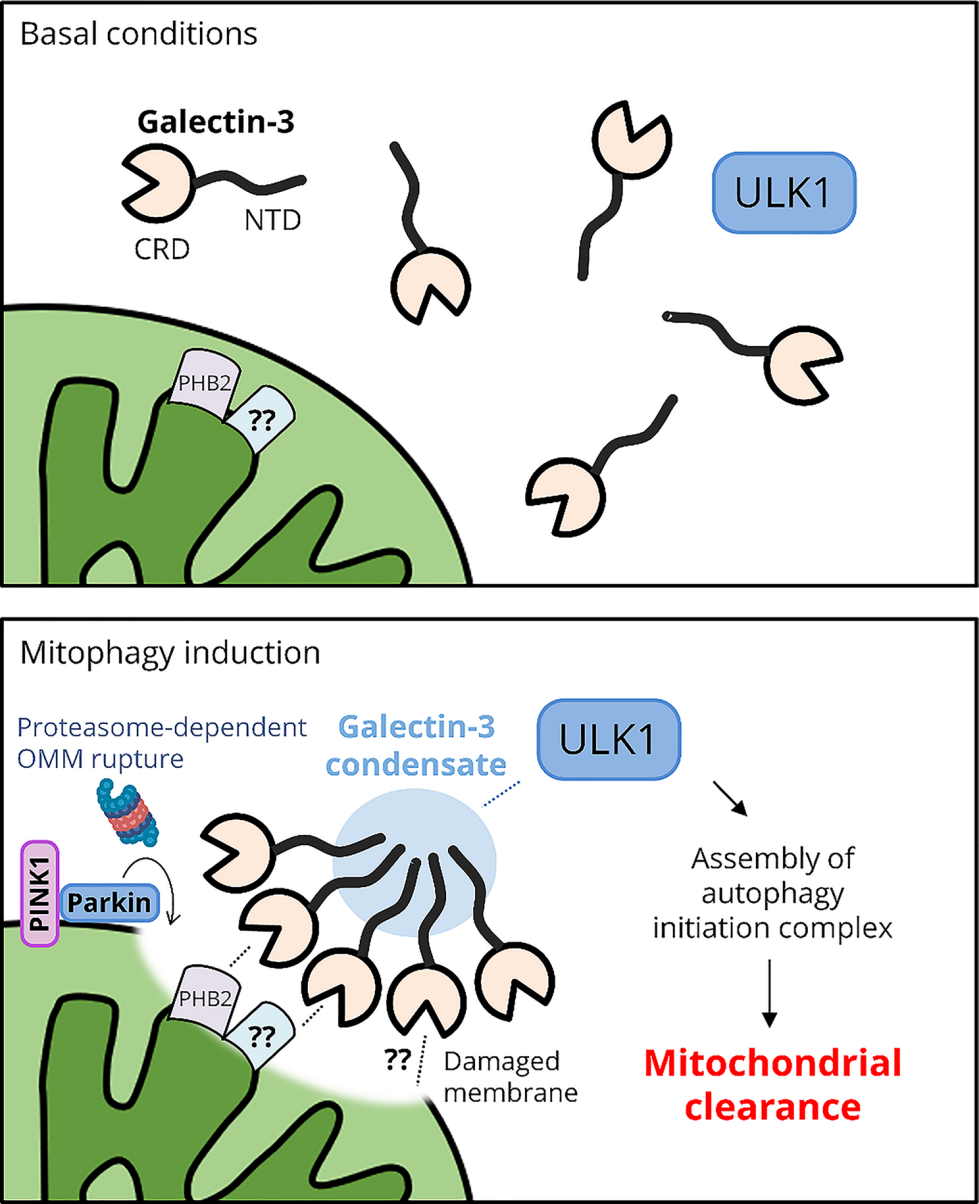
The role of Galectin-3 in sensing OMM rupture and facilitating mitophagy. (Top) In basal conditions, Galectin-3 is cytosolic and does not gain access to the IMM. (Bottom) Upon the activation of PINK1 and Parkin, proteasome-dependent OMM exposes molecular signatures of mitochondrial damage that recruit Galectin-3. Subsequently, Galectin-3 forms condensates through self-association of its N-terminal intrinsically disordered region that is essential for condensate formation around the damaged mitochondria. Subsequently, Galectin-3 condensates promote the recruitment of the autophagy initiation factor ULK1, thereby facilitating autophagic clearance of damaged mitochondria

## Discussion

The fidelity of selective autophagy relies on the accurate targeting of the cargo and localized activation of the autophagic machinery. Previous studies have demonstrated that compromised cellular membranes can trigger various forms of selective autophagy, including the autophagy of the damaged lysosome (lysophagy) and intracellular pathogens undergoing endosomal escape (xenophagy). The molecular signatures of ruptured membranes—including glycoproteins or glycolipids located on the luminal side of endomembranes—act as signals recognized by the cellular machinery responsible for membrane repair, as well as ubiquitylation, proteasomal degradation, and autophagic removal [34, 35]. Although glycan-mediated cargo recognition is not known on the mitochondria, the molecular signature of compromised OMM—including the exposure of IMM protein PHB2, MTFP1, or OPA1—mediates the direct recruitment of LC3-associated membranes to sequester the damaged mitochondria during mitophagy.

In this study, our APEX2-based proximity labeling proteomic study has uncovered Galectin-3 as a novel mitophagy factor essential for mitochondrial quality control (Figs. 1 and 2). Given that mitochondrial dynamics are closely coordinated with quality control mechanisms, we are intrigued to examine whether Galectin-3 contributes to this form of regulation. We immunofluorescently quantified mitochondrial form factor (reflecting network complexity; increased with branching), aspect ratio (indicator of morphology; increased with elongation and decreased with fragmentation), the number of branch junctions (reflecting network connectivity and fusion), and integrated density of mitochondrial signals (representing mitochondrial mass). Galectin-3 depletion did not result in significant changes in these parameters, at least in basal conditions (Fig. S4A and B).

Mechanistically, Galectin-3 is relocalized to the mitochondria, where it interacts with PHB2 and forms structures that encapsulate the perinuclear clustered mitochondria during mitophagy (Fig. 3). The colocalization of Galectin-3 and mitochondria is dependent on proteasome activity as well as Parkin (Fig. 4D–I). These results imply that Galectin-3 is targeted to damaged mitochondria and may function as a sensor of ruptured OMM by interacting with the exposed IMM components. However, the specific ligand responsible for the engagement of Galectin-3 to the IMM during mitophagy remains elusive. In our experiments, assessing the requirement of PHB2 for Galectin-3 recruitment with siRNA knockdown (Fig. S3G–I) has proven challenging as prohibitins are essential for cell proliferation and mitochondrial function. It is possible that PHB2 levels are not a limiting factor for Galectin-3 recruitment, and the residual PHB2 present after siRNA knockdown (Fig. S1F) may still be sufficient to recruit Galectin-3. In addition, other IMM proteins could participate in Galectin-3 recruitment upon OMM rupture. Analysis from the BioGRID proteomic database (https://thebiogrid.org/) indicated that ATP5C1 [40] and SLC25A5 [41] (which is reported to involve in PINK1/Parkin-mediated mitophagy [42]) are candidate Galectin-3-interacting IMM protein, both of which were also present in the PHB2 proximal proteome in our study.

Interestingly, glycosylated isoforms of mitochondrial proteins have been reported, raising the possibility that OMM rupture could expose glycosylation in specific contexts [43]. Thus, we do not dismiss the possibility that Galectin-3 could bind to glycans on the IMM, although the glycosylation patterns of IMM proteins remain currently unknown. Although it is not known whether Galectin-3 can directly interact with a specific type of lipid, a previous study indicated that Galectin-3 can localize to mitochondrial membranes via protein cofactors such as the Ca²⁺/phospholipid-binding annexin A7 (ANXA7/synexin) during stress, suggesting a possible link between lipid recognition and Galectin-3 recruitment to the damaged mitochondria [44]. Thus, additional studies to identify the precise trigger of Galectin-3 during the topological exposure of IMM are warranted.

Experiments using the mutant of Galectin-3 that fails to self-associate through its N-terminal IDR demonstrated that the ability of Galectin-3 to form biomolecular condensates may be critical for mitophagy. The elimination of large, hydrophobic residues (tyrosine and tryptophan) necessary for the formation of biomolecular condensate completely abolished the clustering of Galectin-3 to the vicinity of damaged mitochondria and the mitochondrial clearance (Fig. 6). These data strongly suggest that the formation of biomolecular condensate of Galectin-3 is a driving force of mitophagy. Studies have shown that a number of selective autophagy cargos form biomolecular condensates for efficient autophagy turnover [45, 46]. In yeast, the formation of semi-liquid condensate of the aminopeptidase 1 (Ape1) is critical for its transport to the vacuole through cytoplasm-to-vacuole targeting (Cvt) pathway, a form of selective autophagy [47]. In mammals, the LLPS of p62/SQSTM1 and PGL granules are required for their selective autophagy removal [48–50]. It was proposed that the formation of biomolecular condensate containing selective autophagy receptors would stabilize the selective autophagy receptor-cargo complex via promoting multi-valent interaction and increased avidity. Moreover, the liquidity of the condensate on the cargo recognition site may allow a dynamic regulation of autophagic membrane initiation, in which smaller assemblies of autophagic machinery can be merged into a larger, active isolation membrane initiation site, making efficient autophagosomal membrane assembly [45].

We also found that the Galectin-3 puncta in basal conditions were occasionally co-localize with endoplasmic reticulum (ER) and p62/SQSTM1 bodies (but not PGL granules) (Fig. S5A), indicating that the Galectin-3 may also participate in condensate formation at ER and p62/SQSTM1 and may potentially contribute their autophagic clearance. Upon mitophagy induction with OA, the formation of Galectin-3 condensate is essential for its clustering around the mitochondria, efficient ULK1 recruitment, and mitochondrial clearance (Fig. 6). Together, we propose that Galectin-3 condensate may facilitate efficient assembly of ULK1-containing membrane complexes into an isolation membrane, thereby preparing the damaged, ruptured mitochondria for efficient autophagic clearance (Fig. 7).

A recent study has revealed that Parkin-independent mitophagy occurs during apoptosis, wherein BAX/BAK-mediated OMM permeabilization (MOMP) results in the exposure of IMM proteins [51]. This specific form of mitophagy, referred to as “apoptotic mitophagy”, involves the ubiquitylation of the exposed IMM proteins, which are then recognized by autophagy receptors to eliminate mitochondria undergoing MOMP, thereby attenuating apoptosis. Thus, the capacity of Galectin-3 to recognize damaged OMM suggests that it may also promote apoptotic mitophagy. In support of this hypothesis, early research has demonstrated that in response to apoptotic stimuli, Galectin-3 translocates from the cytosol to mitochondria, inhibiting apoptosis by suppressing cytochrome C release [44]. This result implied that Galectin-3 may broadly senses the mitochondria with compromised OMM integrity.

Interestingly, we note that mitophagy protein BNIP3 (but not its paralog NIX/BNIP3L) levels were consistently and markedly reduced upon the loss of Galectin-3 (Fig. S2B). Given the established role of BNIP3 in hypoxia- and iron depletion-induced mitophagy, this finding suggested that Galectin-3 regulates mitochondrial quality control through mechanisms beyond the canonical PINK1/Parkin pathway.

During PINK1/Parkin-dependent mitophagy, damaged mitochondria form clusters (known as mitoaggregate) in the perinuclear region. This is considered an initial protective mechanism that sequesters the damaged components, limiting their cytotoxicity before autophagic removal. Given the ability of Galectin-3 to agglutinate cells and biomolecules, we suspected that Galectin-3 may function to promote the clustering of OMM-ruptured mitochondria. However, the reconstitution of the WY/G mutant of Galectin-3 in *LGALS3* KO cells did not hinder the aggregation of mitochondria after OA-induced damage (data not shown), suggesting that Galectin-3 is not essential for mitoaggregate formation during mitophagy.

Taken together, our study highlights a distinct role of Galectin-3 in sensing the mitochondria undergoing OMM rupture and facilitating the local recruitment of autophagy initiation proteins ULK1. These results extend our understanding of Galectin-3 beyond endo-lysosomal membrane damage response. Moreover, the APEX2 proximity labeling study also identified several numerous molecules whose enrichment on the OMM rupture site is comparable to that of Galectin-3 during mitophagy. These factors could function coordinately with Galectin-3 to promote localized biogenesis of autophagosomes or augment the recognition of ruptured OMM in response to mitochondrial damage. Future studies aimed at characterizing these additional molecules should provide a more comprehensive understanding of the molecular processes that occur following OMM rupture during PINK1/Parkin-mediated mitophagy.

## Experimental procedures

### Expression plasmids and cloning

PHB2-APEX2-FLAG expressing construct was generated by PCR amplification of human *PHB2* and subcloned to pLenti-IRES-Puro. pEGFP-N1-LGALS3 was a gift from Dr. Fu-Tong Liu (Academia Sinica). Galectin-3-FLAG-expressing construct was generated by cloning human *LGALS3* cDNA to pLenti-IRES-Neo vector. Constructs expressing EGFP-tagged or FLAG-tagged N- (1-121 a.a.), C-terminal (122–250 a.a.), or WY/G mutant (W22G/W26G/Y36G/Y41G/Y45G/Y54G/Y63G/Y70G/Y79G/Y89G/Y101G/Y107G, a gift from Dr. Jie-Rong Huang) of Galectin-3 were generated by Gibson assembly. For the 2xCOX8-EGFP-tagRFP-PEST mitophagy flux reporter, 2xCOX8 and PEST sequence were PCR amplified from mito-SRAI_pcDNA3 (a gift from Dr. Atsushi Miyawaki, RIKEN, Japan) and Gibson assembled with EGFP, tagRFP, and pLenti-IRES-Puro.

### Mammalian cells

HeLa *LGALS3* KO, HeLa *LGALS3* KO/Galectin-3-EGFP, and HeLa Galectin-3 KO with transgenic knock-in of Galectin-3 cDNA at AAVS1 locus are gifts from Dr. Fu-Tong Liu (Academia Sinica, Taiwan). HeLa Parkin cells were generated by stable transfection of pIRES-Hyg3 vector (Clontech) with human Parkin cDNA. HeLa Parkin cells expressing wild-type or mutant Galectin-3 were generated by lentiviral transduction of pLenti-IRES-Neo vector carrying Galectin-3 wild-type or mutant cDNA into HeLa Parkin cells. HeLa Parkin PHB2-APEX2 cells were generated by lentiviral transduction of pLenti-PHB2-APEX2-IRES-Puro and pLenti-PHB1-IRES-Neo into HeLa Parkin cells.

### Chemicals

All chemicals were dissolved in DMSO and stored at -80 °C in small aliquots. The working concentrations of the chemicals are Oligomycin (2.5 µM) together with antimycin A (250 nM) (OA) and epoxomicin (200 nM).

### Antibodies

For immunofluorescence analysis, primary antibodies were mouse rabbit anti-COXIV (Proteintech 11242-1-AP, 1:1000), rabbit anti-COXIV (Abcam ab16056, 1:1000), mouse anti-ATP5B (Millipore MAB3494, 1:1000), anti-FLAG M2 (Sigma F1804, 1:1000), and mouse anti-Parkin (Cell signaling 4211, 1:1000). The secondary antibodies were donkey anti-rabbit IgG AlexaFluor 594 (Invitrogen A21207, 1:750), donkey anti-mouse IgG Alexa Fluor 488 (Invitrogen A21202, 1:750), goat anti-mouse IgG1 AlexaFluor 488 (Invitrogen A21121, 1:750), and goat anti-mouse IgG2b AlexaFluor 488 (Invitrogen A21145, 1:750).

For Western blot analysis, primary antibodies were rabbit anti-ATG5 (Cell signaling 12994, 1:1000), rabbit anti-ATG7 (Sigma, A2856, 1:1000), mouse anti-ATP5A (Proteintech 66037-1-Ig, 1:5000), mouse anti-ATP5B (Millipore, MAB3494, 1:1000), mouse anti-β-actin (Santa Cruz sc-47778, 1:5000), mouse anti-BECN1 (Santa Cruz sc-48341, 1:4000), mouse anti-BNIP3 (Proteintech 68091-1-Ig, 1:5000), mouse anti-BNIP3L/NIX (Proteintech 68118-1-Ig, 1:5000), rabbit anti-COX IV (Abcam ab16056, 1:10000), mouse anti-Flag M2 (Sigma F1804, 1:2000), rabbit anti-Galectin-3 (a gift from Dr. Fu-Tong Liu, GeneTex #90625, 1:5000), mouse anti-GAPDH (Santa Cruz sc-32233, 1:3000), rabbit anti-LC3B (Cell Signaling #3868, 1:1000), mouse anti-Parkin (Cell signaling 4211, 1:1000), rabbit anti-p62/SQSTM1 (Proteintech 18420-1-AP, 1:5000), mouse anti-PHB2 (Santa Cruz sc-133094, 1:10000), mouse anti-ULK1 (Santa Cruz sc-390904, 1:1000). The secondary antibodies were IRDye^®^ 800CW goat anti-mouse IgG (LI-COR 926-32210, 1:7500), IRDye^®^ 680RD goat anti-mouse IgG (LI-COR 926-68071, 1:7500), Peroxidase AffiniPure™ goat anti-mouse IgG (H + L) (Jackson immune Research 115-035-003, 1:20000), and Peroxidase AffiniPure™ goat anti-rabbit IgG (H + L) (Jackson immune Research 111-035-003, 1:10000).

### APEX2 proximity labeling

The experimental procedure was performed according to Han et al. [27] In brief, 1 × 10^8^ HeLa Parkin/PHB2-APEX2 cells were treated with either DMSO or OA for 3.5 h at 37 °C, after which biotin-phenol was added at a final concentration of 0.5 mM. The cells were incubated for an additional 30 min at 37 °C. Subsequently, H_2_O_2_ was added to the medium at 0.5 mM for a 1-min labeling. Subsequently, the cells were washed with the quenching solution (10 mM sodium azide, 10 mM sodium ascorbate, and 5 mM Trolox) and lysed with RIPA buffer (50 mM Tris-HCl pH 7.5, 150 mM NaCl, 1% NP-40, 0.5% sodium deoxycholate, 0.1% SDS) supplemented with 1x cOMPLETE protease inhibitor cocktail (Roche # 04693159001) and a mixture of phosphatase inhibitors (5 mM sodium fluoride, 1 mM sodium orthovanadate, 1 mM sodium pyrophosphate, and 1 mM β-Glycerophosphate). For the purification of biotinylated proteins for proteomic analysis, streptavidin magnetic beads (ThermoFisher #88816), which are pre-washed with RIPA, were added to the cell lysates and rotated at room temperature for 1 h. The mixtures were subjected to two washes with RIPA, one wash with 1 M KCl, followed by one wash with 0.1 M Na_2_CO_3_, and one wash with 2 M urea in 10 mM Tris-HCl (pH 8.0). The samples were finally washed once more with RIPA and boiled in 3x Laemmli buffer supplemented with 2 mM biotin and 20 mM DTT for subsequent label-free proteomic analysis.

### Lentivirus preparation

All pLenti-IRES-Puro- and pLenti-IRES-Neo-based expression plasmids were co-transfected with helper plasmids pCMVΔR8.91 and pMD.G (RNAi Core, Academia Sinica, Taiwan) into HEK293FT cells by using T-Pro Non-liposome Transfection Reagent II (T-Pro Biotechnology, Taiwan). The lentiviral supernatant was collected 48 h after transfection and filtered through a 0.45 μm filter. The target cells were infected in the presence of polybrene at 8 µg/mL. Twenty-four hours post-infection, the viral supernatant was removed and replaced with fresh growth medium containing appropriate antibiotic (0.5 µg/ml puromycin and/or 500 µg/ml G418) and selected for at least two weeks.

### In situ proximity ligation assay (PLA)

HeLa Parkin cells were fixed with 4% paraformaldehyde for 10 min and followed by a 10-min permeabilization in ice-cold methanol at -20 °C. The Duolink^®^ in situ proximity ligation assay was performed according to the manufacturer’s instructions (Sigma-Aldrich) with primary antibodies [rabbit anti-LGALS3 (Proteinech 82024-1-RR, 1:1000) and mouse anti-PHB2 (Santa Cruz sc-133094, 1:2000)].

### Co-immunoprecipitation

For co-immunoprecipitation of Galectin-3 and PHB2, HeLa Parkin cells expressing C-terminal FLAG-tagged Galectin-3 were treated with either DMSO or OA (2.5 µM oligomycin + 250 nM antimycin A) for 4 h. Whole cell lysates were prepared in cell lysis buffer [25 mM HEPES (pH7.4), 150 mM NaCl, 1 mM EDTA], 1x cOMPLETE protease inhibitor cocktail and a mixture of phosphatase inhibitor. Cells were resuspended in the cell lysis buffer and incubated on ice for 20 min prior to a 20-min centrifugation at 16,000 xg. The supernatants were collected, diluted in cell lysis buffer, and then incubated with anti-FLAG M2 antibody (Sigma F1804) at 1:1000 dilution. BSA-blocked protein G Mag Sepharose Xtra (Cytiva #28967070) was incubated with the samples overnight at 4 °C. Finally, the beads were washed three times for 5 min with cell lysis buffer supplemented with AEBSF and analyzed by Western blot.

### Western blot analysis

Cell lysates or immunoprecipitates were boiled in 1x Laemmli buffer containing 2.5% β-mercaptoethanol at 95 °C for 5 min. Samples were separated by SDS-PAGE, transferred to either PVDF or NC membrane, blocked with 5% BSA in TBST (0.1% Tween-20), and incubated with appropriate primary combined with either HRP- or IRDye 800CW/680RD-conjugated secondary antibodies. The signals were visualized by either chemiluminescent substrates and LAS-4000 image system (GE) or Odyssey Imaging System (LI-COR). Band quantification were performed with either ImageJ (HRP conjugates) or Image Studio version 5.2 (IRDye conjugates).

### Mitochondrial fractionation

For the isolation of mitochondrial fractions, HeLa Parkin cells were treated with either DMSO or OA for 4 h. Cells were collected, washed once with ice-cold D-PBS, and resuspended in Buffer A [0.25 M sucrose, 10 mM Tris-HCl (pH 7.5), 10 mM KCl, 1.5 mM MgCl_2_, 1 mM EDTA, 1x cOMPLETE protease inhibitor cocktail]. Cell pellets were incubated on ice for 15 min and homogenized by using 1 mL syringes with 28G needles with 25 strokes. The samples were centrifuged at 700 xg for 5 min at 4 °C, and the supernatants were collected as post-nuclear supernatant (PNS) fractions. For the separation of cytosolic and mitochondrial fractions, PNS was centrifuged at 8000 xg for 30 min at 4 °C. The supernatants (cytosol fraction) were collected, and the pellets (mitochondrial fraction) were washed again with Buffer A, centrifuged, and then resuspended in Buffer A for subsequent analyses.

### Light microscopy

For immunofluorescence staining of cultured cells, samples were fixed with 2% paraformaldehyde for 10 min at room temperature and followed by a 10-min incubation with methanol at -20 °C. The samples were then incubated with a primary antibody for either 1 h or overnight, followed by a secondary antibody incubation for at least 30 min. Finally, the samples were mounted with ProLong Diamond Antifade Mountant (Invitrogen P36971). Fluorescent micrographs were acquired using a Zeiss AxioImager M2 microscope equipped with Apotome 2, an Axiocam 702 mono camera, and a Zeiss PLAN APOCHROMAT 63X/1.4NA Oil DIC objective. Samples that were stained with the same antibodies during the same experiment were captured using the same acquisition time.

### Mitophagy assays

The clearance of damaged mitochondria was evaluated using Western blot analysis to measure the inner membrane protein COXIV or by immunofluorescent staining of ATP5B (mitochondrial matrix protein) followed by ImageJ-based quantitation of ATP5B-positive puncta. For the assessment of mitophagy flux, a plasmid encoding 2xCOX8-EGFP-tagRFP-PEST (which encodes a tandem bifluorescent reporter to detect lysosome-delivered, acidified mitochondria and a C-terminal PEST degron to ensure efficient mitochondrial targeting) was transiently transfected into the HeLa Parkin cells with lipofectamine 2000 (Invitrogen), following the manufacturer’s instruction. The numbers of mitolysosomes (red-only puncta) were quantitated using the “mQC counter” ImageJ script published by Montava-Garriga et al. [52]

### Galectin-3-EGFP analysis

To monitor the localization of Galectin-3, constructs expressing Galectin-EGFP were stably transfected into HeLa Parkin cells. Stable clones with Galectin-EGFP expression levels comparable to endogenous Galectin-3 were selected for analysis. Cells were fixed with 2% paraformaldehyde for 10 min at room temperature and directly mounted. Samples were images by Zeiss AxioImager M2 microscope equipped with Apotome 2 and number of Galectin-3-positive structures was counted in a blinded manner.

### RNAi-mediated knockdown

siRNA-mediated gene knockdowns were performed using Lipofectamine RNAiMAX (Invitrogen #13778150) at a final concentration of 10 nM DsiRNA, according to the manufacturer’s instructions. Forty-eight hours post-transfection, experiments were performed, and gene knockdown efficiency was assessed using Western blot analysis. Dicer substrate siRNAs (DsiRNA) were purchased from IDT DNA. The target sequences of siRNA are as follows:

NC1 (Non-targeting control), 5’-CGUUAAUCGCGUAUAAUACGCGUAT-3’; 3’- CAGCAAUUAGCGCAUAUUAUGCGCAUA-5’;

*PHB2* (hs.Ri. PHB2.13.4), 5’- UUGGGGACAGUGCGUGAUUUCUCAG-3’; 3’- ACAACCCCUGUCACGCACUAAAGAGUC-5’;

For shRNA-mediated knockdown, the virus supernatant at a multiplicity of infection (MOI) of three with 8 µg/ml polybrene was used for the infection. Twenty-four hours after infection, the virus supernatant was replaced with fresh media. The infected cells were selected with puromycin at 0.5 µg/ml (HeLa) or 1.0 µg/ml (SH-SY5Y) 48 h post-infection. Lentiviruses expressing shRNA were obtained from the RNA Technology Platform and Gene Manipulation Core (Academia Sinica, Taiwan). Target sequences of shRNA are as follows:

Control (TRC2.void), 5’-AGTTCAGTTACGATATCATGTCTCGAGACATTCGCGAGTAACTGAACTTTTTTG-3’.

*ATG5* (TRCN0000151963), 5’-CCTGAACAGAATCATCCTTAA-3’.

*LGALS3* #1 (TRCN000029304), 5’- GCTCACTTGTTGCAGTACAAT-3’.

*LGALS3* #2 (TRCN000029306), 5’- GCAAACAGAATTGCTTTAGAT-3’.

*LGALS3* #3 (TRCN000029307), 5’- GCAGTACAATCATCGGGTTAA-3’.

### Statistical analysis

For comparisons of the three or more groups, one-way ANOVA with Kruskal-Wallis test followed by Dunn’s test for multiple comparisons was used for comparisons. For determining the difference between groups of cells with or without mitochondrial-localized Galectin-3, one-way ANOVA with Welch’s test was used. Statistical parameters and significance are included in the Figures and the Figure Legends. All statistical tests were two-tailed.

## Supplementary Information

Below is the link to the electronic supplementary material.

Supplementary Material 1

Supplementary Material 2

## Data Availability

All data are available in the main text and the supporting information.
