# Supplementary material for: Galectin-3 directs mitophagy in response to Parkin-/proteasome-dependent rupture of mitochondrial outer membrane
Source: Biol Direct. 2025 Nov 6;20:108. doi: 10.1186/s13062-025-00692-1 (PMC12590881; doi:10.1186/s13062-025-00692-1)
Supplement: Supplementary file 1 — Supplementary Material 1 [file 13062_2025_692_MOESM1_ESM.pdf]

Figure S1

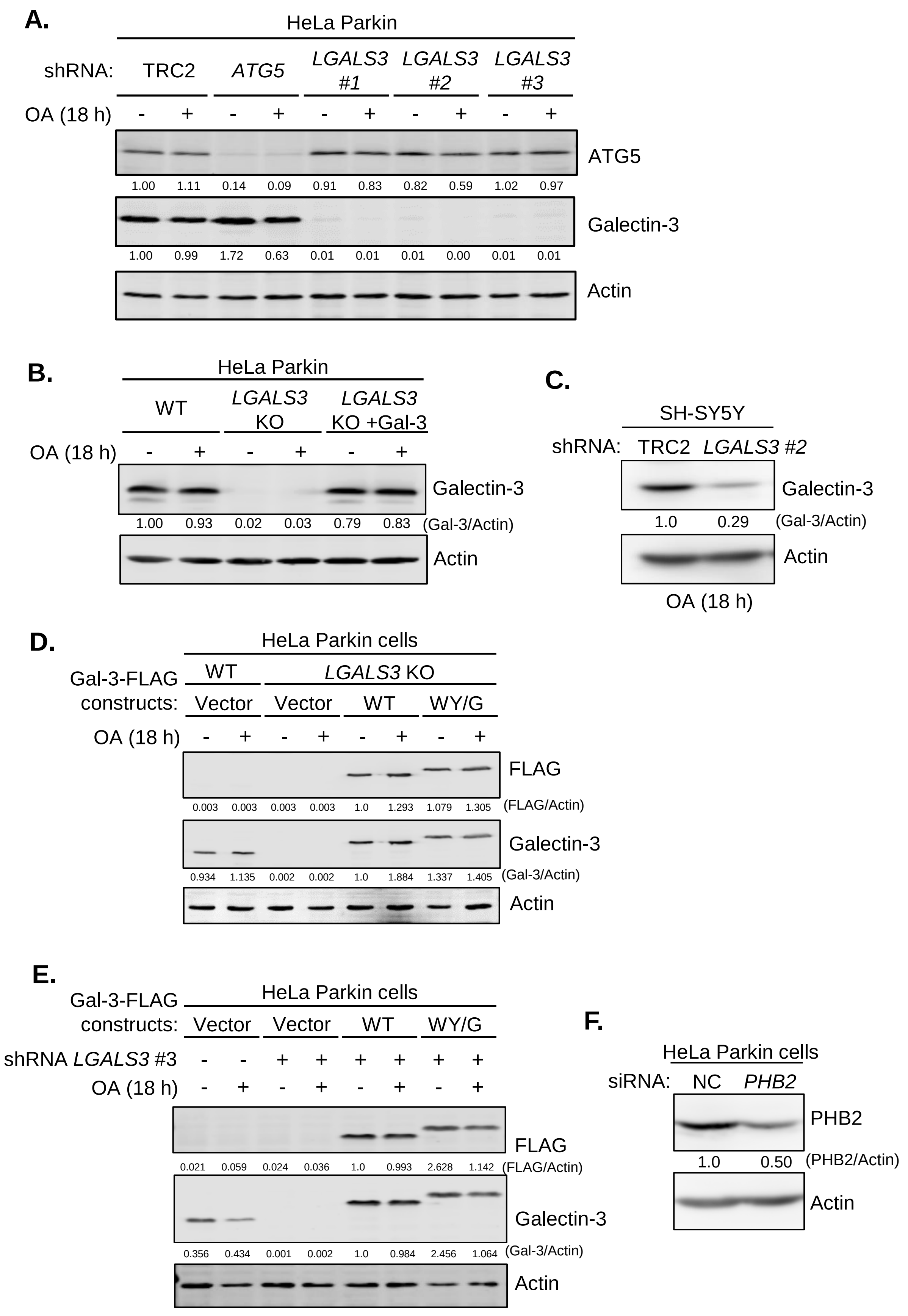
A.
HeLa Parkin
shRNA: TRC2 ATG5 LGALS3 #1 LGALS3 #2 LGALS3 #3
OA (18 h) - + - + - + - + - +
ATG5
1.00 1.11 0.14 0.09 0.91 0.83 0.82 0.59 1.02 0.97
Galectin-3
1.00 0.99 1.72 0.63 0.01 0.01 0.01 0.00 0.01 0.01
Actin
B.
HeLa Parkin
WT LGALS3 KO LGALS3 KO +Gal-3
OA (18 h) - + - + - +
Galectin-3
1.00 0.93 0.02 0.03 0.79 0.83 (Gal-3/Actin)
Actin
C.
SH-SY5Y
shRNA: TRC2 LGALS3 #2
Galectin-3
1.0 0.29 (Gal-3/Actin)
Actin
OA (18 h)
D.
HeLa Parkin cells
Gal-3-FLAG constructs: WT Vector LGALS3 KO Vector WT WY/G
OA (18 h) - + - + - + - +
FLAG
0.003 0.003 0.003 0.003 1.0 1.293 1.079 1.305 (FLAG/Actin)
Galectin-3
0.934 1.135 0.002 0.002 1.0 1.884 1.337 1.405 (Gal-3/Actin)
Actin
E.
HeLa Parkin cells
Gal-3-FLAG constructs: Vector Vector WT WY/G
shRNA LGALS3 #3 - - + + + + + +
OA (18 h) - + - + - + - +
FLAG
0.021 0.059 0.024 0.036 1.0 0.993 2.628 1.142 (FLAG/Actin)
Galectin-3
0.356 0.434 0.001 0.002 1.0 0.984 2.456 1.064 (Gal-3/Actin)
Actin
F.
HeLa Parkin cells
siRNA: NC PHB2
PHB2
1.0 0.50 (PHB2/Actin)
Actin

Figure S2

A.

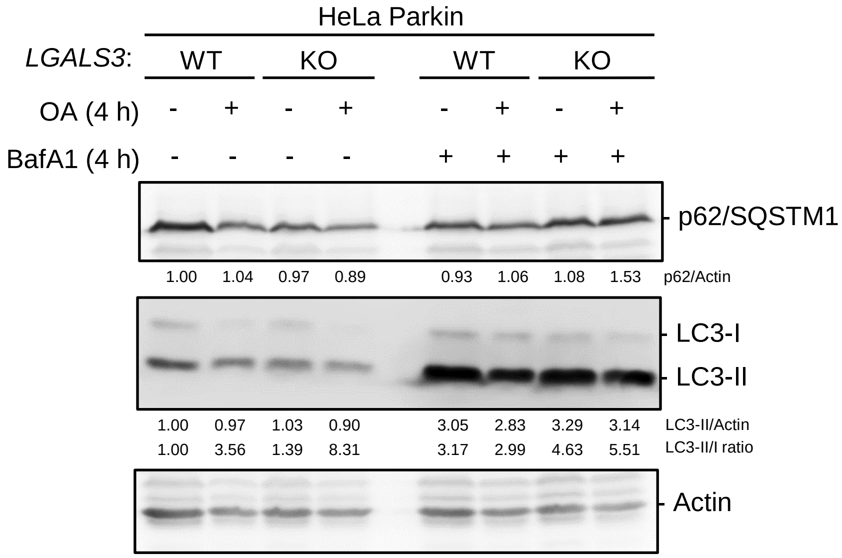

HeLa Parkin
LGALS3:
WT
KO
WT
KO
OA (4 h)
- + - + - + - +
BafA1 (4 h)
- - - - + + + +
p62/SQSTM1
1.00 1.04 0.97 0.89 0.93 1.06 1.08 1.53 p62/Actin
LC3-I
LC3-II
1.00 0.97 1.03 0.90 3.05 2.83 3.29 3.14 LC3-II/Actin
1.00 3.56 1.39 8.31 3.17 2.99 4.63 5.51 LC3-II/I ratio
Actin

B.

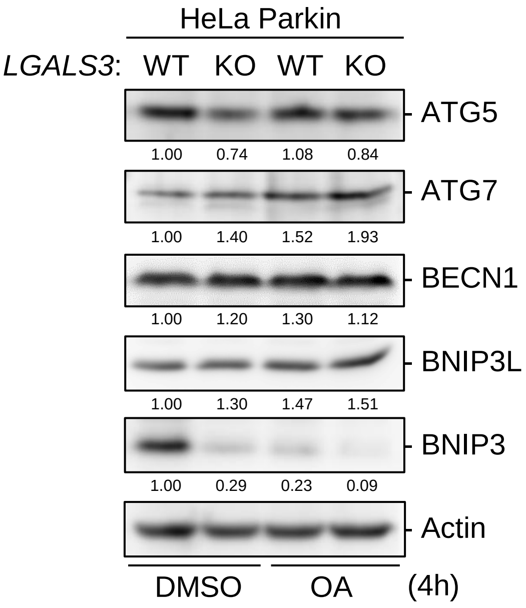

HeLa Parkin
LGALS3: WT KO WT KO
ATG5
1.00 0.74 1.08 0.84
ATG7
1.00 1.40 1.52 1.93
BECN1
1.00 1.20 1.30 1.12
BNIP3L
1.00 1.30 1.47 1.51
BNIP3
1.00 0.29 0.23 0.09
Actin
DMSO OA (4h)

Figure S3

A.

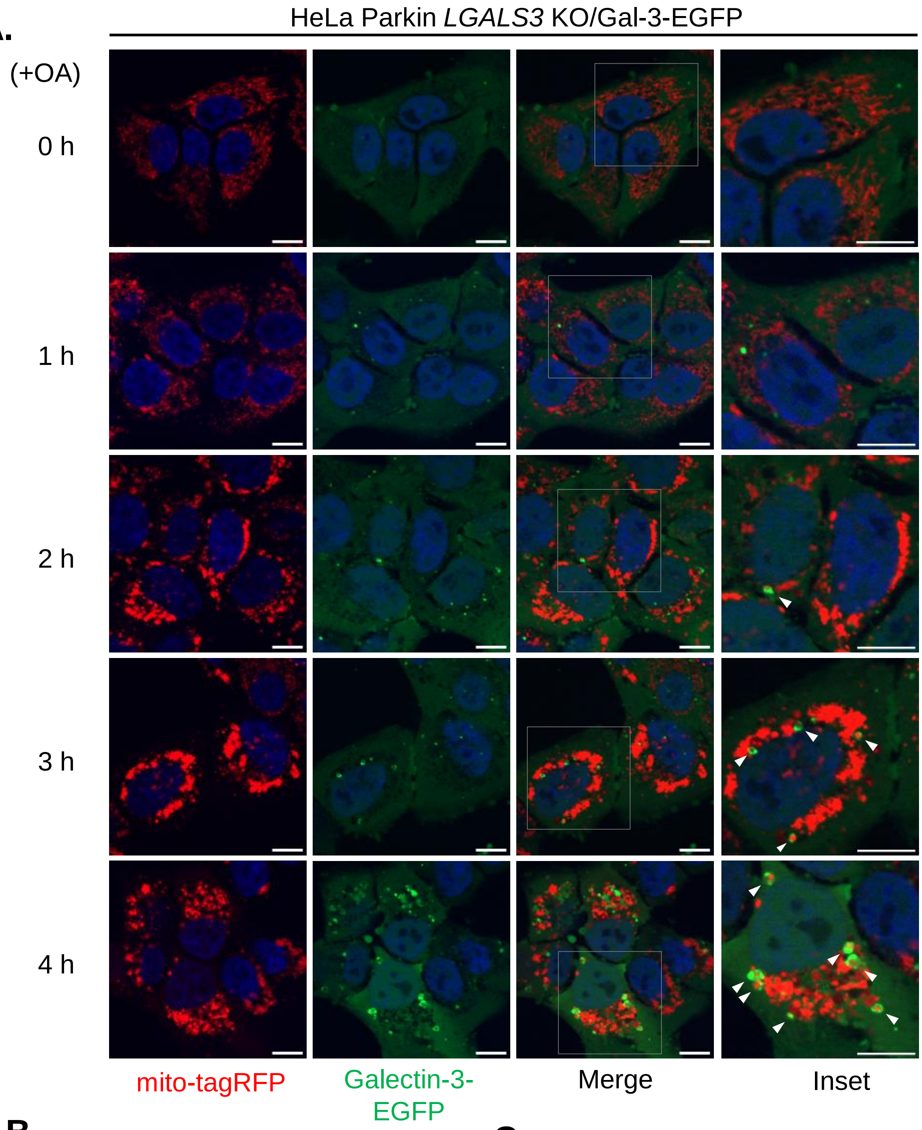

B.

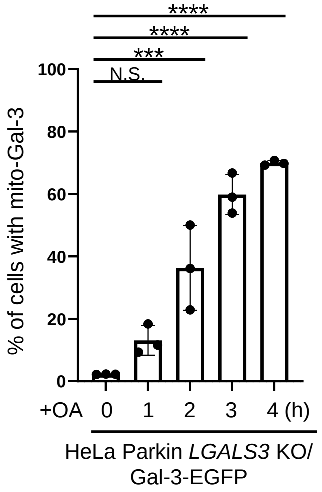

C.

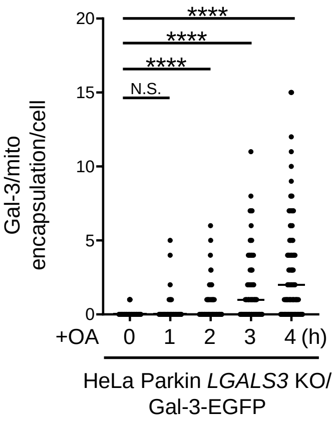

Figure S3, continued

D.

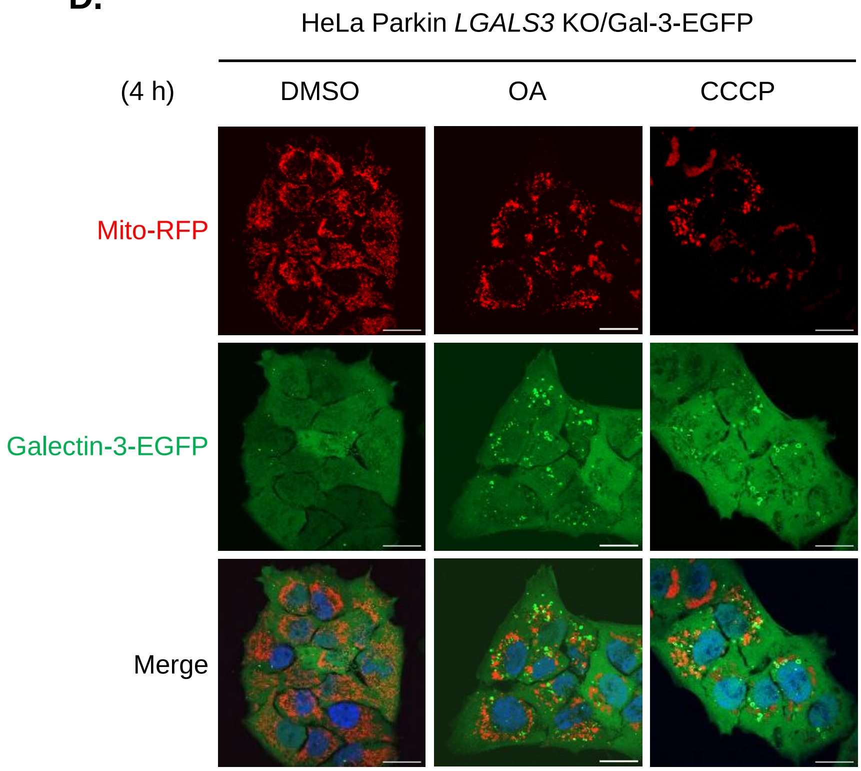

E.

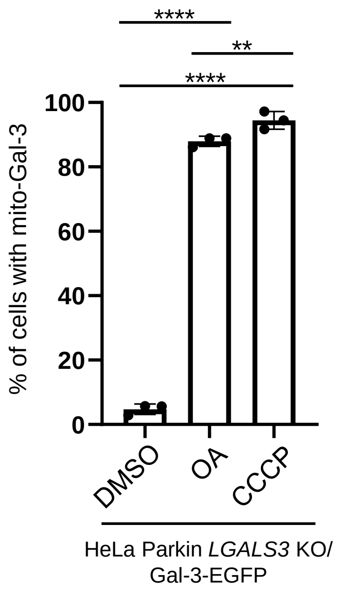

F.

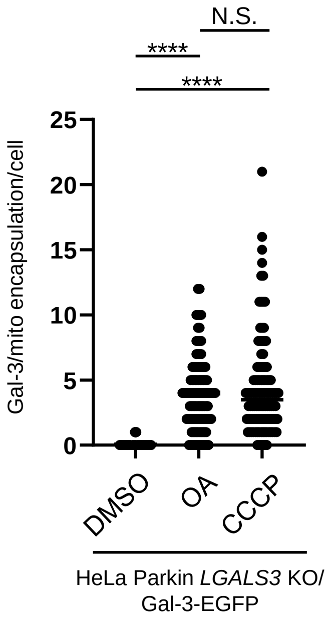

Figure S3, continued

G.

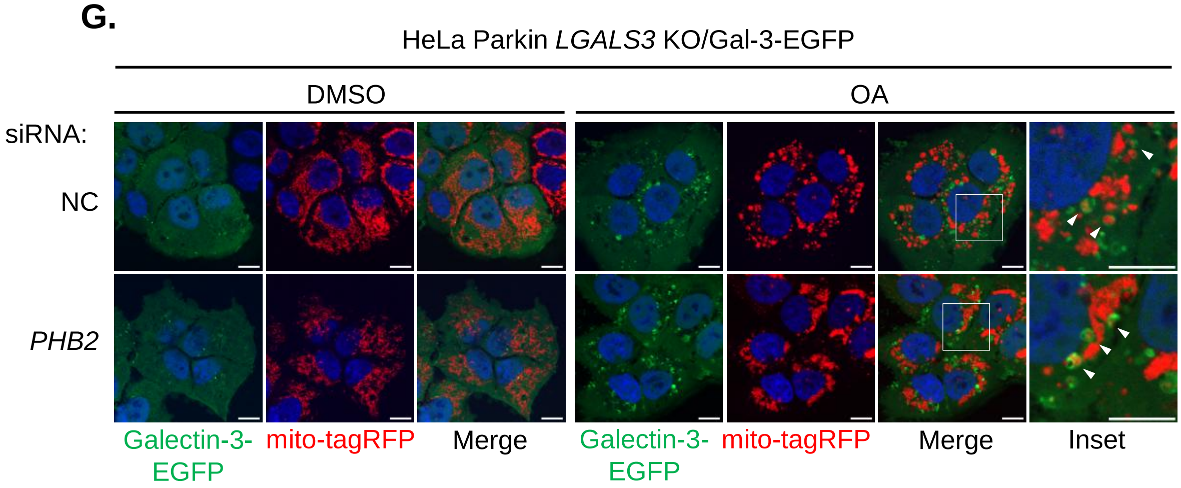

H.

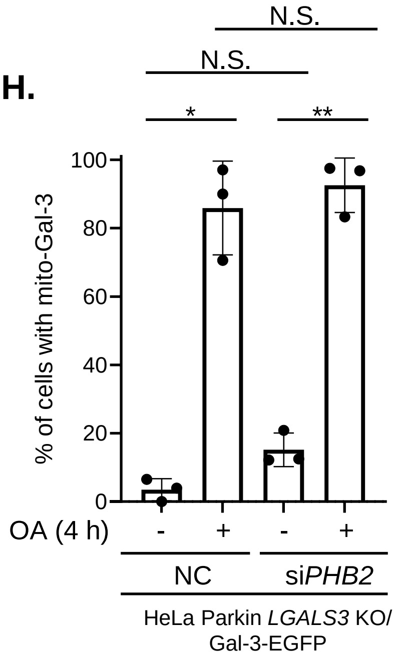

I.

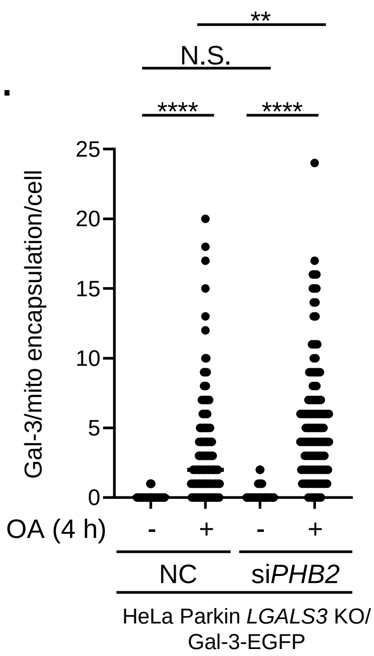

Figure S4

A.

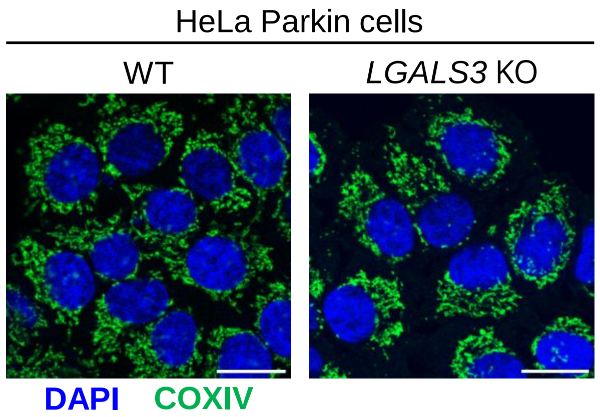
HeLa Parkin cells
WT
LGALS3 KO
DAPI
COXIV

B.

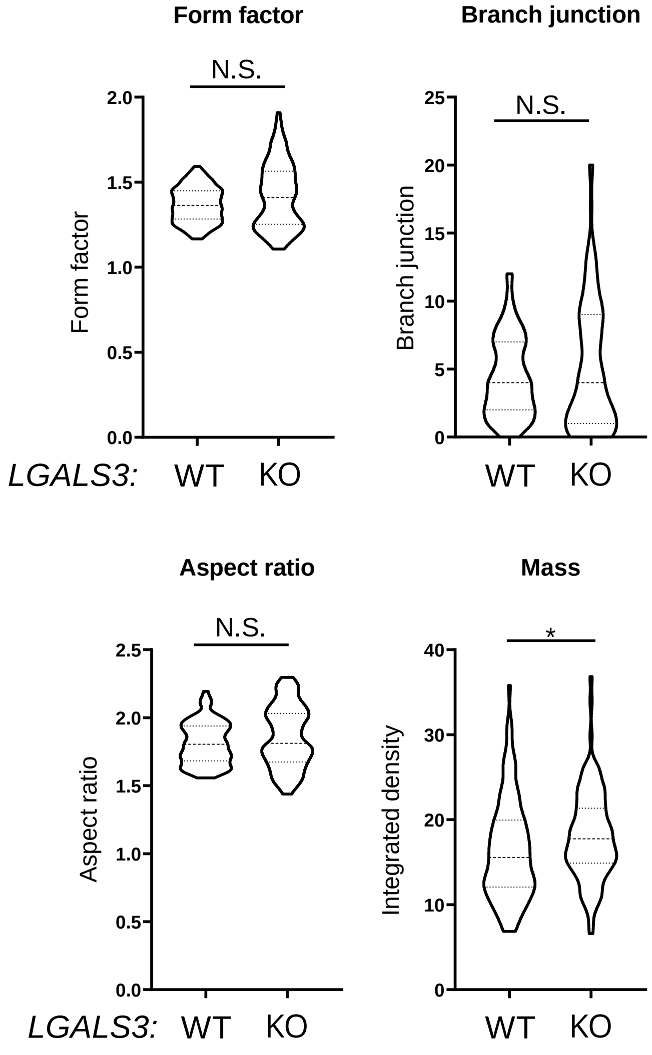
Form factor
N.S.
2.0
1.5
1.0
0.5
0.0
Form factor
LGALS3: WT KO
Branch junction
N.S.
25
20
15
10
5
0
Branch junction
WT KO
Aspect ratio
N.S.
2.5
2.0
1.5
1.0
0.5
0.0
Aspect ratio
LGALS3: WT KO
Mass
*
40
30
20
10
0
Integrated density
WT KO

A.

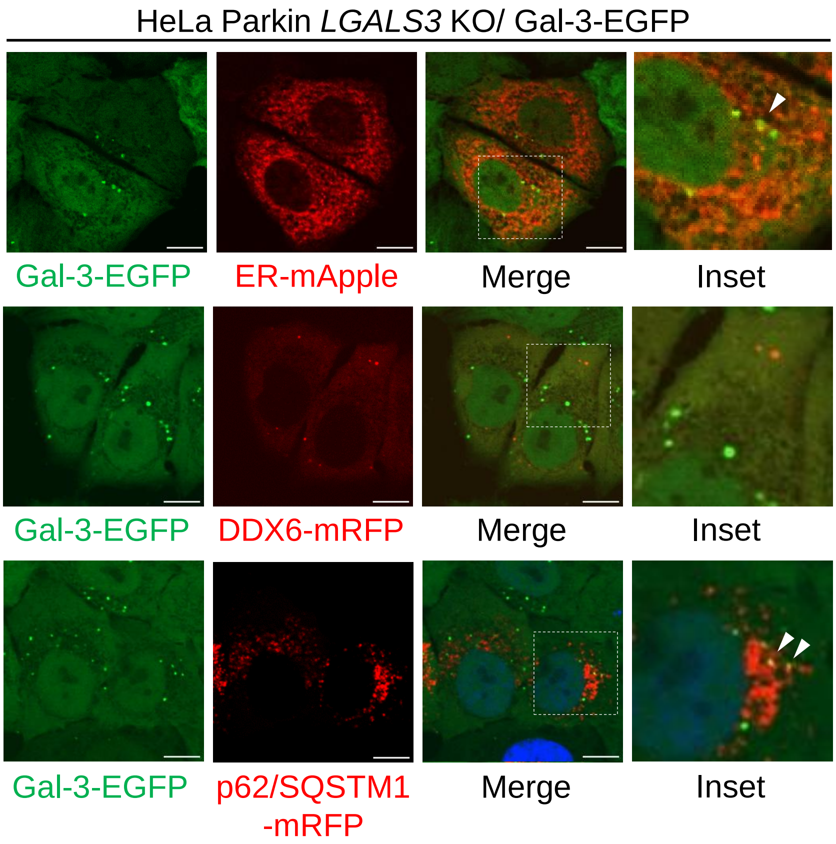
HeLa Parkin *LGALS3* KO/ Gal-3-EGFP
Gal-3-EGFP
ER-mApple
Merge
Inset
Gal-3-EGFP
DDX6-mRFP
Merge
Inset
Gal-3-EGFP
p62/SQSTM1 -mRFP
Merge
Inset

Table S1

Uniprot	Gene Name	Spectra index-OA	Spectra index-DMSO	OA/DMSO	PEP
Q07960	ARHGAP1;CDC42GAP;RHOGAP1	22853050	0	**OA only**	4.13E-14
P12081	HARS;HRS;hCG_1782886	6291005	0	**OA only**	2.35E-08
P23381-1	IFI53;WARS;WRS	6149650	0	**OA only**	3.36E-14
Q16881-1	GRIM12;KDRF;TXNRD1;hCG_1812757	5903550	0	**OA only**	2.09E-10
P22626-1	HNRNPA2B1;HNRPA2B1	5837450	0	**OA only**	1.00E-28
P08133	ANX6;ANXA6;hCG_39152	5755180	0	**OA only**	1.40E-24
P37108	SRP14	5298050	0	**OA only**	1.24E-19
P17655	CANPL2;CAPN2	4942400	0	**OA only**	1.23E-13
Q5TFE4	LP2642;NT5C2L1;NT5DC1;hCG_2031155	4924200	0	**OA only**	5.76E-28
C9J837	RAB3GAP1;KIAA0066;RAB3GAP;hCG_25660	4812010	0	**OA only**	4.90E-14
P42566-1	AF1P;EPS15;RP11-275F13.2-002	4671200	0	**OA only**	3.90E-16
P54105	CLCI;CLNS1A;ICLN	4621750	0	**OA only**	4.54E-14
P53041	PPP5;PPP5C	4478350	0	**OA only**	8.43E-10
P09651-1	HNRNPA1;HNRPA1	4377800	0	**OA only**	4.49E-07
P15311	EZR;VIL2	4129350	0	**OA only**	6.77E-13
P04080	CST6;CSTB;STFB;hCG_401291	3940400	0	**OA only**	6.37E-06
P35573-1	AGL;GDE	3916350	0	**OA only**	1.33E-12
O15371	EIF3D;EIF3S7	3809110	0	**OA only**	3.22E-17
O75886-1	HBP;STAM2	3563000	0	**OA only**	7.38E-16
Q9Y570-1	PME1;PP2593;PPME1;PRO0750	3445550	0	**OA only**	1.95E-11
O75815-1	BCAR3;NSP2;SH2D3B	3343250	0	**OA only**	1.84E-07
B7Z7L3	CYB5R3;DIA1;Z93241.1-006	3297200	0	**OA only**	6.96E-09
P61019	RAB2;RAB2A	3275600	0	**OA only**	3.10E-13
Q96K76-1	USP47	3259200	0	**OA only**	7.69E-12
Q9UNN5-1	CGI-03;FAF1;UBXD12;UBXN3A	3229800	0	**OA only**	5.28E-08
Q9Y263	PLAA;PLAP	3205600	0	**OA only**	1.31E-14
Q9NPQ8-3	RIC8A	3165450	0	**OA only**	2.71E-10
Q6YP21-1	CCBL2;KAT3;RBMXL1;hCG_23341	3159350	0	**OA only**	1.69E-11
P23368	ME2;hCG_23687	3065800	0	**OA only**	3.21E-07
O60271-1	HLC6;HSS;KIAA0516;MAPK8IP4;SPAG9;SYD1;HLC4	2741300	0	**OA only**	7.00E-20
P52209	PGD;PGDH;hCG_2006054;RP4-736L20.2-001	2697050	0	**OA only**	2.39E-08
Q15436	SEC23A	2602000	0	**OA only**	8.56E-11
A2A274	ACO2;RP3-347H13.8-002;ACON	2590500	0	**OA only**	5.27E-08
P14866	HNRNPL;HNRPL;P/OKcl.14	2545700	0	**OA only**	4.71E-07
Q05086-1	E6AP;EPVE6AP;HPVE6A;UBE3A;hCG_18679	2498000	0	**OA only**	8.73E-08
F4ZW65	DRBF;ILF3;MPHOSPH4;NF90	2441950	0	**OA only**	1.81E-11
Q5TDH0-3	DDI2	2425650	0	**OA only**	4.15E-06
P50213-1	IDH3A	2420520	0	**OA only**	1.05E-06
Q9UL15-2	BAG5;KIAA0873	2371950	0	**OA only**	4.52E-23
Q15750	MAP3K7IP1;TAB1;hCG_41717;RP3-407F17.2-002	2299100	0	**OA only**	1.11E-11
A6NDG6	PGP	2286595	0	**OA only**	3.86E-11
Q5VZK9-1	CARMIL;LRRC16;LRRC16A	1871135	0	**OA only**	4.88E-07
Q7LGA3-1	HS2ST;HS2ST1;KIAA0448	1842200	0	**OA only**	6.64E-08
A5PKX8	USP19;KIAA0891;ZMYND9	1671080	0	**OA only**	4.51E-06
O14974-1	MBS;MYPT1;PPP1R12A	1668340	0	**OA only**	6.35E-10
Q9UHD1-1	CHORDC1;CHP1	1609050	0	**OA only**	1.66E-08
O95672-1	ECEL1;UNQ2431/PRO4991;XCE	1414825	0	**OA only**	2.86E-12
Q12768	KIAA0196	1362385	0	**OA only**	2.45E-11
Q6P2H3-1	CCDC21;DKFZp434P232	1337770	0	**OA only**	2.71E-06
Q9UL25	KIAA0118;RAB21	1230625	0	**OA only**	2.18E-06
Q9Y2A7-2	HEM2;KIAA0587;NAP1;NCKAP1	1219195	0	**OA only**	1.32E-06
D4HL82	TRIP12;KIAA0045	1210885	0	**OA only**	1.66E-08
Q9BXF6	GAF1;KIAA0857;RAB11FIP5;RIP11;DKFZp434H018	1169250	0	**OA only**	3.32E-06
Q9NT62-1	ADAM15;MDC15	862980	0	**OA only**	9.59E-08
P40121	RFC1;RFC140;LLDBP	610520	0	**OA only**	4.92E-07

(continued on the next page)

Table S1, continued

Uniprot	Gene Name	Spectra index-OA	Spectra index-DMSO	OA/DMSO	PEP
P62820-1	RAB7;RAB7A	29253500	592935	**49.34**	2.98E-91
Q13492-1	APG3;APG3L;ATG3	8923450	391445	**22.80**	2.80E-08
**O60260-1**	**LGALS3;MAC2**	**16768500**	**789700**	**21.23**	**2.14E-08**
Q14103-1	AFCP;CAPG;MCP	10698250	554050	**19.31**	5.81E-10
O15111	INPPL1;SHIP2	7832150	556700	**14.07**	2.58E-21
Q15181	RAB1;RAB1A;hCG_22592;RAB1C;RAB1B	12955550	946400	**13.69**	2.27E-13
Q96KA5-1	CALM;PICALM;KIAA0656;SNAP91	2697900	236025	**11.43**	9.24E-06
Q9Y697-1	PARK2;PRKN	26131000	2392100	**10.92**	1.47E-39
Q02790	AUF1;HNRNPD;HNRPD	5528000	522100	**10.59**	5.75E-11
P55072	IOPPP;PP;PPA1	6944800	691050	**10.05**	5.37E-08
P12277	CLPTM1L;CRR9	12211750	1216350	**10.04**	1.41E-08
Q8NFF5-1	HUSSY-08;NFS1;NIFS;hCG_38220;RP1-309K20.1-003	4679400	493850	**9.48**	6.23E-07
P32754	FKBP4;FKBP52	6511400	706900	**9.21**	1.50E-82
Q13057-2	BIRC6;KIAA1289	7003150	762050	**9.19**	2.45E-09
P34932	VCP;DKFZp434K0126	279100000	33442000	**8.35**	3.99E-192
P82673-1	CKB;CKBB	12070000	1490250	**8.10**	1.42E-21
B4DLV7	HPD;PPD	6596450	895500	**7.37**	1.77E-10
P19338	COASY;PSEC0106	7289050	1120100	**6.51**	3.64E-10
B4DVE7	APG2;HSPA4;HS24/p52;HS24/P52	7597400	1169500	**6.50**	5.98E-47
P82673-1	HDCMD11P;MDS023;MRPS28;MRPS35;PSEC0213	3865550	595450	**6.49**	9.41E-08
P26639	TARS	10816450	1711000	**6.32**	7.44E-11
B4DLV7	GDI2;RABGDIB	31165000	4978000	**6.26**	8.50E-38
P19338	NCL;hCG_33980	4823550	791750	**6.09**	1.49E-06
B4DVE7	ANX11;ANXA11;hCG_22492;RP11-369J21.10-001	51968000	8547200	**6.08**	1.40E-57
P37802	CDABP0035;KIAA0120;TAGLN2	51069500	8876800	**5.75**	6.09E-35
P11413-2	G6PD	16826000	2935850	**5.73**	1.14E-25
P20073-1	ANX7;ANXA7;OK/SW-cl.95;SNX	62492000	11125200	**5.62**	6.59E-36
P18206-1	VCL;RP11-178G16.3-002	15632250	2916050	**5.36**	5.56E-37
P41240	CSK;hCG_40804	2748150	522150	**5.26**	4.21E-07
O14964-1	HGS;HRS	19728200	3758850	**5.25**	5.46E-29
B4DPD5	DKFZp564E242;HSPC263;OTB1;OTU1;OTUB1	4116800	794000	**5.18**	4.42E-19
O15067	KIAA0361;PFAS;hCG_31283	3802600	736750	**5.16**	1.22E-08
Q9Y371-1	CGI-61;KIAA0491;SH3GLB1	7041750	1406250	**5.01**	1.72E-08
O00203-1	ADTB3A;AP3B1	3324100	673300	**4.94**	4.35E-07
P17987	CCT1;CCTA;TCP1	28431500	5807700	**4.90**	3.67E-37
O60547	GMDS;RP1-118B18.1-002	4338550	919150	**4.72**	8.97E-09
Q12800-1	LSF;SEF;TFCP2	2994190	641800	**4.67**	2.32E-93
Q92783-1	STAM;STAM1	12243000	2628550	**4.66**	4.70E-45
Q15645-1	TRIP13	41170500	9030700	**4.56**	2.39E-44
Q06124-1	PTP2C;PTPN11;SHPTP2;BPTP-3	4501575	990710	**4.54**	1.86E-14
B7Z5E3	LDHA;PIG19;hCG_96677	34812500	7795150	**4.47**	3.55E-16
B2RBD5	TUBB3;TUBB4;hCG_1983504	21545100	4885300	**4.41**	2.73E-200
Q9BPX3	CAPG;NCAPG;NYMEL3	35827500	8157400	**4.39**	4.23E-55
P30520	ADSS;ADSS2;RP11-518L10.4-002	13143500	3057700	**4.30**	2.78E-15
P17812	CTPS	27685500	6588600	**4.20**	6.31E-33
P0CG48	UBC; ubiquitin	361970000	86522000	**4.18**	1.24E-67
Q6PJG6-1	C7orf27	6840800	1656350	**4.13**	1.00E-23
Q13200	PSMD2;TRAP2;DKFZp564A2282	22372000	5497650	**4.07**	1.25E-47
Q7Z6Z7-1	HUWE1	3951250	987020	**4.00**	1.07E-18
B7Z809	MTHFC;MTHFD;MTHFD1	17340500	4338650	**4.00**	3.01E-24
