# Supplementary material for: Galectin-3 directs mitophagy in response to Parkin-/proteasome-dependent rupture of mitochondrial outer membrane
Source: Biol Direct. 2025 Nov 6;20:108. doi: 10.1186/s13062-025-00692-1 (PMC12590881; doi:10.1186/s13062-025-00692-1)
Supplement: Supplementary file 2 — Supplementary Material 2 [file 13062_2025_692_MOESM2_ESM.docx]

**Supplementary Figure Legends**

**Supplementary Figure 1. Verification of protein expression and depletion.** Western blot analysis of **(A)** HeLa Parkin cells expressing control shRNA (TRC2) or shRNA targeting *LGALS3* or *ATG5* incubated with DMSO or OA for 18 h, **(B)** HeLa Parkin wild-type (WT), Galectin-3 knockout (*LGALS3* KO), or Galectin-3 knockout with single-copy knock-in of *LGALS3* (*LGALS3* KO +Gal-3) treated with either DMSO or OA for 18 h, **(C)** SH-SY5Y expressing *LGALS3* shRNA, **(D)** HeLa Parkin *LGALS3* KO cells reconstituted with empty vector (Vector), wild-type (WT), or WY/G mutant of Galectin-3 treated with either DMSO or OA for 18 h, **(E)** HeLa Parkin cells expressing control (TRC2) or *LGALS3* shRNA and reconstituted with shRNA non-targeted (NTm) constructs encoding wild-type (WT) or WY/G mutants of Galectin-3 followed by a treatment with either DMSO or OA for 18 h, and **(F)** HeLa Parkin cells treated with Control (NC) or *PHB2* siRNA. Band intensities were quantified by densitometry and normalized to loading controls (actin).

**Supplementary Figure 2. Western blot analysis of autophagy- and mitophagy-related proteins in Galectin-3 KO cells (A)** Western blot analysis of HeLa Parkin wild-type (WT) and *LGALS3* knockout (KO) cells treated with DMSO or OA for 4 h in the presence or absence of 200 nM bafilomycin A1 (BafA1). **(B)** Western blot analysis of HeLa Parkin WT and *LGALS3* KO cells treated with DMSO or OA for 4 h. Normalized quantification (with actin) of band intensities is shown below each panel.

**Supplementary Figure 3. (A)** Representative images of HeLa Parkin *LGALS3* KO cells expressing Galectin-3-EGFP and mito-tagRFP treated with OA from 0 to 4 h. **(B)** Quantification of the percentage of cells with Galectin-3 encapsulating the mitochondria (mito-Gal-3). One-way ANOVA with Welch’s test. **(C)** Quantitation of Galectin-3-positive structures encapsulating the mitochondria per cell. One-way ANOVA with Kruskal-Wallis test. **(D)** Representative images of HeLa Parkin *LGALS3* KO cells expressing Galectin-3-EGFP and mito-tagRFP treated with DMSO, OA, or CCCP for 4 h. **(E)** Quantification of the percentage of cells with Galectin-3 encapsulating the mitochondria. One-way ANOVA with Welch’s test. **(F)** Quantitation of Galectin-3-positive structures encapsulating the mitochondria per cell. One-way ANOVA with Kruskal-Wallis test. **(G)** Representative images of HeLa Parkin *LGALS3* KO cells expressing Galectin-3-EGFP. Cells were treated with either control (NC) or PHB2 siRNA and subjected to either DMSO or OA for 4 h before immunofluorescent analysis. Arrows indicate the mitochondria encapsulated by Galectin-3. **(H)** Quantification of the percentage of cells with Galectin-3 encapsulating the mitochondria. One-way ANOVA with Welch’s test. **(I)** Quantitation of Galectin-3-positive structures encapsulating the mitochondria in per cell basis. One-way ANOVA with Kruskal-Wallis test. N.S. non-significant, ***P*<0.01, ****P*<0.001, *****P*<0.0001 for all tests. Scale bars, 10 µm.

**Supplementary Figure 4.**  **Analysis of mitochondrial dynamics and mass in Galectin-3 KO cells.**  **(A)** Representative images of HeLa Parkin wild-type (WT) and *LGALS3* KO cells were stained for COXIV. **(B)** Quantitative assessment of mitochondrial morphology and network organization in HeLa Parkin wild-type (WT) and *LGALS3* KO cells. Mitochondrial form factor (reflecting network complexity; increased with branching), aspect ratio (indicator of morphology; increased with elongation and decreased with fragmentation), the number of branch junctions (reflecting network connectivity and fusion) per mitochondrion, and integrated density of COXIV signals (representing mitochondrial mass per cell) were measured. Welch’s t-test. N.S. non-significant, **P*<0.05. Scale bars, 20 µm.

**Supplementary Figure 5.**  **(A)** Immunofluorescence analysis of Galectin-3–EGFP puncta under basal conditions in HeLa Parkin cells co-expressing Galectin-3–EGFP with ER-mApple (ER marker), DDX6-mRFP (PGL marker), or p62-mRFP (p62/SQSTM1 bodies). Scale bars, 10 µm.

**Supplementary Table 1.** Proteomic data of the list of candidate PHB2 interacting proteins shown in Table 1. A filter of OA/DMSO ratio >4 and PEP <1x10^5^ was applied. The normalized spectral index (which takes account of both protein length and the number of unique peptides per protein) indicates the abundance of the candidates in DMSO- or OA-treated samples. OA/DMSO ratio indicates the ratio of spectral index of OA- and DMSO-treated samples. Galectin-3 is highlighted in Red; other autophagy-/mitophagy-related proteins are highlighted in blue.
